# Endophytic fungi and their secondary metabolites in Qin medicine plants: a comprehensive review of diversity, function, and application

**DOI:** 10.7717/peerj.20487

**Published:** 2026-01-09

**Authors:** Bo-Yang Chen, Tong Li, Wen-Pu Shi, Juan-Juan Yang, Yang Bai, Qi-Meng Xue, Chen-Li Jiao, Pei-Feng Wei, Liang-Liang Chen

**Affiliations:** 1 School of Pharmacy, Shaanxi University of Chinese Medicine, Xianyang, China; 2 The Second Clinical Medical School, Shaanxi University of Chinese Medicine, Xianyang, China; 3 National Drug Clinical Trial Institute, The Second Affiliated Hospital of Shaanxi University of Chinese Medicine, Xianyang, China; 4 School of Biomedicine, Beijing City University, Beijing, China

**Keywords:** Application progress, Biological activity, Endophytic fungi, Qin Medicine, Secondary metabolites, Traditional Chinese herbs

## Abstract

Qin medicine represents the premier local traditional Chinese medicinal herbs in Shaanxi province (China) and its neighboring areas. Endophytic fungi, an essential element of the internal ecosystem of medicinal plants, have attracted considerable attention for their roles in enhancing plant resistance to pests and diseases, increasing the concentration of bioactive compounds, and stimulating plant growth and development. This paper presents the first comprehensive review of endophytic fungi in Qin medicinal plants, summarizing their diversity, effects on plant growth and medicinal quality, as well as novelty and bioactivity of their secondary metabolites. It also highlights their potential applications in promoting plant growth. Furthermore, this study explores the current opportunities and challenges in the research of endophytic fungi within Qin medicinal plants, with the objective of offering a unique perspective for the advancement and development of Qin medicinal plants.

## Introduction

Qin medicine refers to genuine medicinal materials, specifically Dao-di herbs, originating from Shaanxi Province and adjacent regions. The term “Qin” denotes the ancient Qin State, and its geographic scope encompasses the northern Qinling Mountains, stretching west from Xi’an along the central Silk Road to areas in the upper Yellow River basin (Tang et al., 2024a). Traditional Chinese medicine is a life science deeply rooted in the soil of Chinese traditional culture. Currently, the academic community has not yet provided a clear definition for the concept of Qin medicine (Hu et al., 2018). In this paper, Qin medicine is defined as “a traditional medical system that originated and developed within the geographical environment of the Three Qin regions”. It can thus be understood that Qin medicine is a regional traditional medicine and an integral part of traditional Chinese medicine (Tang et al., 2024c).

Qin medicine encompasses a variety of notable species, including Qin Pi, Qin Jiao, the “Top Ten Qin Medicines”, and the “Taibaiqi medicine” (Tang et al., 2024a; Tang et al., 2024b). In 2020, the Shaanxi Administration of traditional chinese medicine (TCM) officially announced a list of 45 “Qin medicine” varieties, comprising 15 primary traditional Chinese herbs, 10 region-specific specialty herbs, and 20 advantageous Chinese patent medicines (Tang et al., 2024a). Table 1 summarizes the basic information of the Qin medicinal plants.

**Table 1:** Key information on Qin medicine and its source plants.

Qin medicine	Chinese name	Source plants	Genus (family)	Medicinal parts	Traditional applications	References
*Fraxini Cortex*	秦皮 (Qinpi)	*Fraxinus rhynchophylla* Hance/*F. chinensis* Roxb./*F. szaboana* Lingelsh/*F. stylosa* Lingelsh	Oleaceae (Oleaceae)	Dried bark	Anti-inflammatory, Antibacterial	Zheng et al. (2024)
*Gentianae Macrophyllae*	秦艽 (Qinjiao)	*Gentiana macrophylla* Pall./*G. straminea* Maxim./*G. crassicaulis Duthie ex* Burk./*G. dahurica* Fisch.	Gentiana (Gentianaceae)	Dried roots	Anti-inflammatory and Analgesic	Zhang et al. (2018a)
*Rheum palmatum*	大黄 (Dahuang)	*Rheum palmatum* L./*R. tanguticum Maxim*. ex Balf./*R. officinale* Baill.	Polygonaceae (Rheum)	Root and rootstock	Anti-inflammatory, Antioxidant, Antibacterial and antiviral	You et al. (2013)
*Astragali Radix*	黄芪 (Huangqi)	*Astragalus membranaceus* (Fisch.) Bge. var. *mongholicus* (Bge.) Hsiao/*A. membranaceus* (Fisch.) Bge.	Astragalus (Fabaceae)	Dried root	Immunomodulation, Cardiovascular Protection	Li et al. (2023)
*Bupleurum chinense*	柴胡 (Chaihu)	*Bupleurum chinense* DC/*B. scorzonerifolium* Willd.	Bupleurum (Apiaceae)	Dried root	Antipyretic and Anti-inflammatory, Sedative and Antiviral	Cheng et al. (2022b)
*Corydalis yanhusuo*	延胡索 (Yanhusuo)	*Corydalis yanhusuo* W. T. Wang	Corydalis (Papaveraceae)	Dried tubers	Analgesic, Sedative	Wu et al. (2023)
*Salvia miltiorrhiza*	丹参 (Danshen)	*Salvia miltiorrhiza* Bge.	Salvia (Lamiaceae)	Dried root	Cardiovascular Protection, Anticoagulant	Chen et al. (2021c)
*Aconitum carmichaeli Debx*	附子 (Fuzi)	*Aconitum carmichaclii* Debx.	Aconitum (Ranunculaceae)	Lateral root	Cardiotonic (for heart failure), Anti-inflammatory and Analgesic	Chen et al. (2021e)
*Eucommia ulmoides*	杜仲 (Duzhong)	*Eucommia ulmoides* Oliv.	Eucommia (Eucommiaceae)	Dried bark	Antihypertensive, Strengthens Bones and Muscles	Liang et al. (2024)
*Gastrodia elata*	天麻 (Tianma)	*Gastrodia elata* Blume	Gastrodia (Orchidaceae)	Underground corms	Sedative and Anticonvulsant, Cardiovascular Protection	Yang et al. (2024b)
*Scutellaria baicalensis*	黄芩 (Huangqin)	*Scutellaria baicalensis* Georgi	Scutellaria (Lamiaceae)	Root	Antibacterial and Antiviral, Anti-inflammatory and Antioxidant	Feng & Cui (2024)
*Cornus officinalis*	山茱萸 (Shanzhuyu)	*Cornus officinalis* Sieb. et Zucc.	Cornus (Cornaceae)	Ripening and dry fruits	Hypoglycemic, Renal Protection	Zhao et al. (2020)
*Polygonatum sibiricum*	黄精 (Huangjing)	*Polygonatum sibiricum* Red./*P. cyrtonema* Hua/*P. kingianum* Coll. et Hemsl.	Polygonatum (Asparagaceae)	Dried rhizomes	Antioxidant and Anti-aging, Hypoglycemic and Lipid-lowering	Zhao et al. (2023)
*Polygala tenuifolia*	远志 (yuanzhi)	*Polygala tenuifolia* Willd/*P. sibirica* L.	Polygala (Polygalaceae)	Root	Memory Improvement and Anti-dementia, Sedative and Anxiolytic	Shen et al. (2024)
*Physochlaina infundibularis*	华山参 (Huanshanshen)	*Physochlaina infundibularis Kuang*	Physochlaina (Solanaceae)	Root	Sedative and Hypnotic, Antiasthmatic	Wang et al. (2023a)
*Gynostemma pentaphyllum*	绞股蓝 (Jiaogulan)	*Gynostemma pentaphyllum* (Thunb.) Makino	Gynostemma (Cucurbitaceae)	Whole grass	Lipid-regulating, Anti-fatigue	Ma et al. (2015)
*Ziziphus spinosa* Semen	酸枣仁 (Suanzaoren)	*Ziziphus jujuba var. spinosa* (Bunge) Hu ex H. F. Chow.	Ziziphus (Rhamnaceae)	dried seeds	Sedative and Hypnotic, Anxiolytic	Hu et al. (2018)
*Asarum sieboldii* Miq.	华细辛 (Huaxixin)	*Asarum sieboldii* Miq.	Asarum (Aristolochiaceae)	Whole grass	Analgesic and Anti-inflammatory, Antipyretic	Wang et al. (2022)
*Astragalus complanatus*	沙苑子 (Shayuanzi)	*Astragalus complanatus Bunge*	Astragalus (Fabaceae)	Dried seeds	Hepatoprotective, Improves Eyesight	
*Rubia cordifolia*	茜草 (Qiancao)	*Rubiae Radix et Rhizoma*	Rubia (Rubiaceae)	Roots and rhizomes	Hemostatic, Antitumor	Feng et al. (2021)
*Forsythia suspensa*	连翘 (Lianqiao)	*Forsythia suspensa* (Thunb.) Vahl	Forsythia (Oleaceae)	Air-dried fruits	Antibacterial and Antiviral, Anti-inflammatory and Antipyretic	Zhang, Wei & Wang (2012)
*Sinopodophyllum hexandrum*	桃儿七 (Taoerqi)	*Sinopodophyllum hexandrum* (Royle) T. S. Ying	Sinopodophyllum (Berberidaceae)	Root and rootstock	Antitumor, Immunomodulation	Bi et al. (2013)

Endophytic fungi are microorganisms that extensively inhabit healthy plant tissues without inducing pathogenicity. In nature, endophytic fungi having evolved a specialized mutualistic relationship with their plant hosts through extended coevolution (Jia et al., 2016). On one hand, the diversity and spatial arrangement of endophytic fungi are strongly shaped by the host plant’s genetics, life cycle phase, and external environmental factors. On the other hand, these fungi contribute critically to plant development by boosting stress resistance and aiding in the generation of secondary metabolites.

Endophytic fungi, as a key component of mycobiome of medicinal plants, have exhibited a substantial correlation with the quality of Chinese herbs (Wang et al., 2023b; Feng et al., 2024). These fungi synthesize bioactive compounds that stimulate the production of pharmacologically active substances in host plants, enhancing their growth and stress tolerance. Notably, some endophytes produce secondary metabolites identical to those of their hosts, making the exploration of these compounds and plant-fungal interactions a key research focus with significant potential for alleviating medicinal plant resource shortages and improving cultivation quality (Wang et al., 2023b).

Unlike existing literature, this study moves beyond the limitations of single-species case studies, this is the comprehensive review of research concerning endophytic fungi related with the Qin medicinal plants. It systematically investigates the diversity of these fungi, their influence on plant growth and development, and the quality of medicinal materials. Furthermore, this study reviews the diversity and biological activities of secondary metabolites produced by these fungi, while discussing their potential applications in plant growth promotion. The paper also addresses the opportunities and challenges inherent in the study of endophytic fungi within the context of Qin medicine, with the aim of providing a novel perspective for the advancement and development of this field. This review is intended for researchers in medicinal plant development, natural product research, and agricultural biotechnology. While using Qin medicine as a regional case study, it reveals universally applicable strategies in plant-microbe interactions and bio-active compound development, providing valuable references for research on diverse medicinal systems and crop species.

## Survey Methodology

A comprehensive literature search was conducted across multiple databases, including Google Scholar, Web of Science, SciFinder, PubMed, CNKI, and Wanfang Data. The search strategy utilized a combination of subject headings and free-text terms, with core keywords comprising “Qin medicine”, “Qin medicinal plants”, and the names of various Qin medicinal plants listed in Table 1 (*e.g.*, *Astragalus membranaceus*, *Rheum palmatum*), all of which were combined with the term ”endophytic fungi”. Literature on Qin medicine plant endophytic fungi published up to August 2024 was utilized for this review, to exclude non-peer-reviewed content such as conference abstracts and preprints.

**Figure 1:**
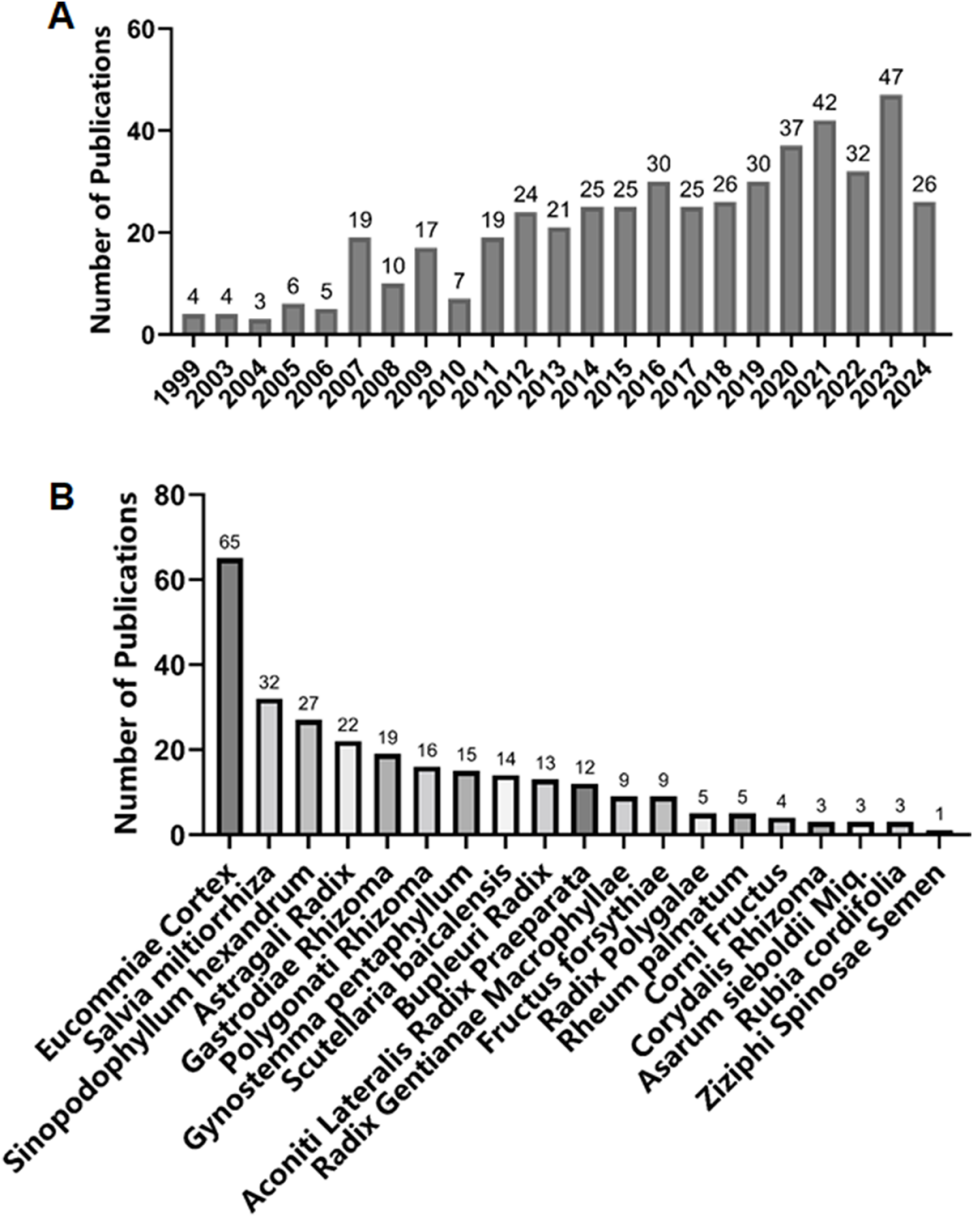
Statistics of reported endophytic fungi in Qin medicine plants. (A) Temporal trends in the number of reported endophytic fungi in Qin medicine plants over different years. (B) The distribution of reported endophytic fungi across various Qin medicine plant species.

Figure 1 summarizes the literature reports on endophytic fungi associated with Qin medicine. According to the publication year data (Fig. 1A), the earliest report on endophytic fungi of Qin medicine was published in 1999, detailing the isolation of endophytic fungi from *Sinopodophyllum hexandrum* (*S. hexandrum*). Since 2010, there has been a gradual increase in terms of the count of reports on endophytic fungi in Qin medicinal plants. Based on the search results for host plants (Fig. 1B), endophytic fungi from *Eucommia ulmoides* (*E. cortex*), *Salvia miltiorrhiza* (*S. miltiorrhiza*), and *Astragali Radix* (*A. Radix)* were the most frequently reported. In contrast, no related endophytic fungi from *Fraxini Cortex, Physochlaina infundibularis* (*R. physochlainae*), and *Astragalus complanatus* were reported due to the limited availability of these plant resources. The literature search in this study may be subject to bias, as less-studied species and unresearched species could be underrepresented due to limitations in database search strategies. Future studies should specifically target these neglected species to fill knowledge gaps.

## Diversity of Endophytic Fungi in Qin Medicinal Plants

Two principal methodologies are utilized to explore the diversity of endophytic fungi in plants: high-throughput sequencing and tissue isolation. High-throughput sequencing offers a relatively comprehensive and effective approach for characterizing endophytic fungal communities (Huo et al., 2020; Kumar & Prasher, 2022). In contrast, tissue isolation focuses on culturable endophytic fungi, which represent only a portion of the total endophytic fungal population, as many species remain uncultivable. Notwithstanding this limitation, the acquisition of culturable endophytic fungi is essential for investigating their biological activities, secondary metabolites, and potential applications. This paper synthesizes the findings from endophytic fungi obtained *via* tissue isolation.

The diversity of endophytic fungal communities residing in Qin medicinal plants were primarily characterized at the genus level through gene sequencing and morphological analysis, with only a limited number of novel species reported. For example, Li et al. isolated a new species of endophytic fungi, *Phlebiopsis xuefengensis* sp. nov. WS01, from the tubers of *Gastrodia elata* (*G. elata*). Zhang et al. (2024) identified ten endophytic fungal isolates from the rhizomes of *Polygonatum sibiricum* (*P. Rhizoma*), including three novel species: *Neosetophoma endophytica*, *N. polygonatum*, and *N. qimenensis*. Table 2 shows a detailed overview of the endophytic fungal species identified in Qin medicinal plants, including representatives from the genera *Fusarium*, *Aspergillus*, *Penicillium*, and *Alternaria*. These fungal species exhibit diverse biological functionalities, representing a pivotal research domain in medicinal plant endophyte studies (Wang et al., 2023b).

**Table 2:** Endophytic fungi of Qin medicinal plants.

Qin medicinal plants	Endophytic fungi at the genus level	References
*Gentiana macrophylla*	*Leptosphaeria, Fusarium, Plenodomus, Septoria, Botrytis, Paecilomyces, Cladosporium, Phaeoseptoria, Hymenoscyphus, Pezicula, Mucor, Didymella, Alternaria, Aspergillus niger*	Cheng et al. (2022a); Luo et al. (2018); Zhang et al. (2018b)
*Rheum palmatum*	*Trichoderma, Penicillium, Fusarium, Colletotrichum, Pestalotiopsis, Periconia, Sarocladium, Cladosporium, Paramyrothecium*	Ren (2024); You et al. (2013)
*A. Radix*	*Fusarium, Bionectria, Cladosporium, Acremonium, Penicillium, Aspergillus, Rhizoctonia, Rhizopus, Hemileia, Gibberella, Ascobolus, Pseudogymnoascus*	Ren et al. (2022)
*Bupleurum chinense*	*Penicillium, Aspergillus, Candida, Alternaria, Bipolaris, Fusarium, Mucor, Cladosporium, Acremonium, Geotrichum, Rhizoctonia, Cephalosporium*	Gao et al. (2012); Li, Luo & Zhang (2015)
*Corydalis Rhizoma*	*Clonostachys, Fusarium, Monilochaete, Alternaria, Leptosphaeria, Mucor, Aspergillus, Penicillium, Arcopilus, Chaetomium, Nectria, Trichoderma, Auricularia, Rhizopus, Eurotiomycetes*	Ma (2016); Wu et al. (2023)
*S. miltiorrhiza*	*Aspergillus, Penicillium, Candida, Trichoderma, Cephalosporium, Verticillium, Botrytis, Nigrospora, Tolypocladium, Alternaria, Curvularia, Cladosporium, Fusarium, Phomopsis, Ascochyta, Sclerotium, Rhizoctonia, Sporidiobolus, Mucor*	Ji et al. (2011b)
*Aconitum Lateralis Praeparata*	*Mucor, Nigrospora, Acremonium, Cladosporium, Alternaria, Trichoderma, Aspergillus, Pleiochaeta, Humicola, Cephaliophora, Penicillium, Botrytis, Candida, Monocillium, Fusarium, Pestalotiopsis, Sphaeropsis, Rhizoctonia, Phacidium, Chaetomium, Rhizopus, Pireella, Cunninighamella*	Li et al. (2009)
*E. Cortex*	*Alternaria, Fusarium, Botrytis, Oidium, Paecilomyces, Phoma, Tubercularia, Pythium, Erysiphe, Cercospora, Rhizoctonia, Tolyposporium, Doassansia, Apiotrichum, Debaryomyces, Leucosporidium, Kernia, Rhodotorula, Aspergillus, Penicillium,*	Ma et al. (2007); Sun (2005); Zhang (2003)
*Gastrodiae Rhizoma*	*Alternaria, Fusarium, Colletotrichum, Phomopsis, Mucor, Epicoccum, Leptosphaeria, Nectria, Pseudocercospora, Trichoderma, Coniothyrium*	Mo et al. (2008)
*Scutellaria baicalensis*	*Fusarium, Minimedusa, Acremonium, Cylindrocladium, Alternaria, Latendraea, Arbusculina, Sclerotium, Asteromella, Acremonium, Colletotrichum, Paecilomyces, Amerosporium, Cladorrhinum, Dendrodochium, Isaria, Myriococcum, Rhizoctonia, Aspergillus, Beverwykella, Bullera, Cryptostroma, Echinocondrium, Gonatobotrys, Libertella, Metarhizium, Mucor, Naemospora*	Cui et al. (2007); Li et al. (2010)
*Cornus officinalis*	*Alternaria, Ascochyta, Botryosphaeria, Colletotrichum, Fusarium, Helminthosporium, Leptosphaerulina, Marssonia, Peyronellaea, Phoma, Phyllosticta, Simplicillium, Talaromyces, Mycelia sterilia*	Zhao et al. (2020)
*P. Rhizoma*	*Sterile mycelia, Fusarium, Aspergillus, Penicillium, Diaporthe, Alternaria, Talaromyces, Colletotrichum, Metapochonia, Arthrinium, Phomopsis, Rhizopus, Cladosporium, Clonostachys,* Sordariomycetes*, Neurospora, Gliocladiopsis, Setophoma,* Ascomycet*e, Kohlmeyeriopsis, Trichoderma, Gibberella*	Cheng et al. (2021); Li et al. (2013)
*Polygala tenuifolia*	*Fusarium, Alternaria, Exserohilum, Aspergillus, Colletotrichum, Diaporthe, Myrothecium, Leptosphaerulina, Cladosporium, Purpureocillium, Phoma, Paraphoma, Rhizopus*	Wei et al. (2021)
*Gynostemma pentaphyllum*	*Fusarium, Leptosphaeria, Microascus, Systemma theiss, Monilia, Chaetomium, Aspergillus, Cylindrocarpon, Phoma, Acremonium, Cladosporium, Alternaria, Didymaria, Cephalosporium, Rhizoctonia, Ascochyta, Cercospora, Aureobasidium, Verticillium, Ceratocystis, Pestalotiopsis*	Ma et al. (2015)
*Rubia cordifolia*	*Fusarium, Trichoderma, Colletotrichum, Cercospora, Alternaria, Penicillium, Mucor, Emmia, Myrothecium, Chaetomium, Plectosphaerella, Phomopsis, Nigrospora, Pestalotiopsis, Botryosphaeria, Neofusicoccum, Bipolaris, Curvularia, Talaromyces, Aspergillus, Ceratobasidium, Trametes, Funalia, Lentinus, Phlebia, Bjerkandera, Phanerochaete, Phlebiopsis, Irpex*	Feng et al. (2021)
*Forsythia suspensa*	*Rhizoctonia, Sclerotium, Microsporum, Helminthosporium, Absidia, Endogone, Cunninghamella, Botryosporium, Syncephalastrum*	Yang, Li & Liu (2007)
*S. hexandrum*	*Aspergillus, Alternaria, Phoma, Chaetomium, Mucor, Trichoderma, Penicillium, Monilia, Fusarium, Rhizoctonia, Pullularia, Periconia, Hormodendrum,*	Bi et al. (2013); Li, Zeng & Zhang (1999); Zhu et al. (2015)

Endophytic fungi extensively colonize various tissues of host plants, forming close mutualistic symbiotic relationships. However, their distribution across different tissues is not uniform, showing significant variations in both quantity and species composition. This spatial distribution pattern is primarily influenced by two factors: first, the specific structure of plant tissues and their localized microenvironments shape the colonization preferences of endophytes; second, environmental differences in plant habitats also markedly affect endophytic fungal composition, which will be discussed in ‘The spatiotemporal dynamics of endophytic fungi’. Table 3 presents endophytic fungal counts from different Qin medicinal plant parts. It should be noted, however, that these data only reflect the overall isolatable fungi in existing studies, without distinguishing dominant strains or precisely quantifying distribution patterns. This limitation arises because: (1) some studies failed to specify the exact tissue origins of isolated fungi; (2) most reports focused on analyzing endophytes in medicinal parts while neglecting systematic investigation of other tissues. With advances in research techniques, scholars have begun examining endophytic fungal distributions at microscopic scales. For instance, Zhao et al. (2007) demonstrated through histological observations that fungal hyphae fragments in *S. hexandrum* rhizomes were not randomly distributed but specifically enriched in the cork layer and secondary phloem. Moreover, localized colonization only occurred within particular microdomains of cortical and secondary phloem cells. These findings highlight the spatial specificity of endophytic colonization and provide insights into the intricate interactions between endophytes and their hosts.

**Table 3:** Enumeration of endophytic fungi from different parts of Qin medicinal plants.

Qin medicine	Root	Stems	Leaf	Other	References
*Gentiana macrophylla*	13	5	4	79 (Unknown)	Cai et al. (2023); Cheng et al. (2022a); Hou et al. (2022); Li et al. (2022); Luo et al. (2018); Xu (2008); Zhang et al. (2018b)
*Rheum palmatum*	14	2	/	/	Chen et al. (2021a), You et al. (2013)
*A. Radix*	131	35	6	/	Gong et al. (2014); Kong (2011); Zheng et al. (2017); Zhou et al. (2012b)
*Bupleurum chinense*	100	167	66	/	Du et al. (2023); Gao et al. (2012); Li, Luo & Zhang (2015)
*Corydalis Rhizoma*	109	114	196	1807 (tuber)	Ma (2016); Wu et al. (2023)
*S. miltiorrhiza*	586	992	84	41 (flower), 9 (Seed)	Bu et al. (2016); Ding (2017); Ji et al. (2011b); Li et al. (2016b); Liu et al. (2020); Ming et al. (2012); Wang et al. (2013); Wang (2020); Zhao (2020); Zhou (2021)
*Aconitum Lateralis Praeparata*	93	59	23	15 (flower)	Li et al. (2009); Zhang (2015)
*E. Cortex*	87	156	95	237 (bark), 40 (Seed)	Chen et al. (2011); Li (2007a); Li (2023); Liang et al. (2014); Ma et al. (2007); Shen et al. (2008); Yang et al. (2012a); Zhang (2003); Zhu (2005)
*Gastrodiae Rhizoma*	/	7	3	7 (flower), 4 (Seed), 57 (tuber)	Liang et al. (2020); Mo et al. (2008); Yang et al. (2024a); Yu et al. (2023)
*Scutellaria baicalensis*	330	264	291	6 (flower)	Cui et al. (2007); Cui et al. (2022); Ji et al. (2011a); Li et al. (2010); Wei et al. (2012)
*Cornus officinalis*	23	166	29	3 (flower), 14 (fruit)	Gao (2019); Zhao et al. (2020)
*Polygonatum sibiricum*	190	9	5	25 (Unknown), 3 (fruit)	Cao et al. (2023); Cheng et al. (2021); Han et al. (2023); Wang et al. (2010)
*Polygala tenuifolia*	51	70	30	85 (Unknown), 18 (above-ground part)	Cui, Guo & Fan (2007); Fu & Xiong (2012); Shen et al. (2024); Wang et al. (2009); Wei et al. (2021)
*Gynostemma pentaphyllum*	13	20	43	/	Fan (2012); Shang et al. (2011); Zhou et al. (2007)
*Ziziphus spinosa Semen*	20	15	5	/	Gao et al. (2014)
*Asarum sieboldii* Miq.	/	/	/	5 (Unknown)	Bao (2012)
*Rubia cordifolia*	/	/	/	42 (Unknown)	Feng et al. (2021)
*Forsythia suspensa*	/	20	19	10 (fruit), 2 (flower)	Niu et al. (2018); Yang, Li & Liu (2007); Zhang, Wei & Wang (2012)
*S. hexandrum*	846	861	224	103 (tuber), 21 (Roots, stems and leaves)	Bi et al. (2013); Li, Zeng & Zhang (1999); Liu et al. (2011b); Wang et al. (2017a); Wang et al. (2006); Yang et al. (2003); Zhou, Li & Ma (2023); Zhu et al. (2015)

## The Spatiotemporal Dynamics of Endophytic Fungi

Variations in regional environments create distinct growth conditions for plants, which can subsequently influence the diversity of endophytic fungi within the same plant species across different habitats. Studies on endophytic fungi of *Rheum palmatum* (*R. palmatum*) (Chen et al., 2021b), *Astragalus mongholicus* (Li et al., 2023), *Corydalis yanhusuo* (Wu et al., 2023), *S. miltiorrhiza* (Wang et al., 2013), *E. cortex* (Dong et al., 2021; Yang et al., 2019; Zhang et al., 2021), *G. elata* (Mo et al., 2008) and *S. hexandrum* (Zhou, Li & Ma, 2023) have demonstrated that the diversity and abundance of endophytic fungi and Operational Taxonomic Units (OTUs) vary significantly across different geographical origins. Geography plays a key role in shaping endophytic fungal communities, with each region harboring distinct taxa. In other words, endophytic fungal strains derived from distinct plant sources demonstrate both conserved functional properties and host-specific adaptations. The growth environment of the host plant significantly influences the composition of the internal microbial community. Cui et al. (2022) found that the endophytic fungal community of *Scutellaria baicalensis* (*S. baicalensis*) exhibit greater similarity when the plants have closer geographical genetic affinity. Additionally, Their analysis identified a statistically significant relationship between *Alternaria* colonization levels and the accumulation of key flavonoids (baicalin, wogonoside, wogonin, and oroxylin A).

The same plant species from the same origin can exhibit seasonal variations in their endophytic fungal communities. Liang et al. (2014) found that the diversity and evenness of endophytic fungi in *Eucommia* bark differ across different habitats. Notably, the diversity of endophytic fungi in *Eucommia* bark is particularly rich in January and November, whereas March and July are characterized by a prominent dominant bacterial community with high dominance and relatively low species richness. Fan (2015) found that the diversity of endophytic fungi in Aconitum exhibits dynamic changes throughout its growth cycle. Endophytic assemblages inhabiting Aconitum root systems Aconitum fluctuated significantly between January and April, showing marked differences compared to May and June. The richness index reached its lowest point in July and remained relatively stable from July to October, with no significant differences among these months. Additionally, the community structure of endophytic fungi changed noticeably from January to May, with substantial compositional differences. In contrast, from June to October, the community structure was more consistent, exhibiting higher similarity and fewer changes (Fan, 2015). Yang et al. (2012a), Yang et al. (2012b) and Liang et al. (2024) reported significantly greater density and α-diversity of bark endophytes isolated from Eucommia in autumn compared to spring.

The age or growth stage of the host plant is an significantly factor influencing the diversity of endophytic fungi. Yao et al. (2023) found that *E. cortex* harbors a core endophytic fungal community, and the prevalence of dominant endophytic fungal genera varies with ages. Additionally, the species composition of these dominant genera undergoes seasonal succession. Tree age is the key factor responsible for differences in the structure and diversity of the endophytic fungal community in *E. cortex* within the same environment (Yao et al., 2023). Wei et al. (2021) reported that the community structure of endophytic fungi in *Polygalae Radix* changes with the plant’s growth duration. Specifically, the highest number of endophytic fungi were isolated from three years old plants, and the richness index, diversity index, and dominance index of endophytic fungi in these plants were also the highest. Additionally, literature reports indicate that the content of primary pharmacological components is highest in three-year-old *Polygalae Radix*, further suggesting that the quality of *Polygalae Radix* is closely associated with its endophytic fungal community. Han et al. (2023) found that the isolation rate of endophytic fungi in the rhizomes of *P. Rhizoma* did not differ significantly between spring and summer. However, Comparative analysis revealed enhanced endophytic fungal diversity in spring, with significant differences in species assemblages between seasons. Cui et al. (2007) and Li et al. (2010) reported that, regardless of whether the wild or cultivated plants of *S. baicalensis*, the quantity of endophytic fungal strains in the mature stage was significantly higher than in the juvenile stage.

More than a few studies have demonstrated that human cultivation practices can significantly influence the diversity of endophytic fungi in Qin medicinal plants. Wang et al. (2009) found that, compared to cultivated *Polygala tenuifolia* (*R. Polygalae*) Willd, wild plants exhibited greater numbers of endophytic fungal strains, broader species distribution, higher counts of active strains, and wider antibacterial spectra. These findings indicate that the species diversity of endophytic fungi in the wild plants of *R. Polygalae* Willd is substantially richer than in cultivated varieties. Traditionally, wild herbs are generally perceived to be of higher quality compared to cultivated herbs. Whether the diversity of endophytic fungi contributes to this quality difference remains an area warranting further investigation.

## Endophytic Fungi and Qin Medicine Ingredients

### Endophytic fungi mediate biosynthesis and accumulation of active pharmaceutical ingredients in Qin medicinal plants

Endophytes can mimic the host’s metabolic pathways, enabling them to produce, induce, and modify bioactive compounds within the host. Through coevolution, particular endophytic fungi have formed characteristic symbiotic partnerships with host plants, directly mediating biosynthetic pathways of pharmacologically active compounds and subsequently determining the therapeutic value of plant-derived medicines (Wang et al., 2023c). Multiple studies on endophytic fungi in Qin medicinal plants have confirmed that the bioactive compounds of Qin medicine are directly associated with specific endophytic fungi.

### Effects of endophytic fungi on active ingredient accumulation in Qin medicinal plants

Numerous studies have indicated that there are significant correlations between the accumulation of active ingredients in various Qin medicinal plants and their endophytic fungi. For instance, Hou et al. (2022) found that the content of loganic acid in *Gentiana officinalis* and *Gentiana siphonantha* was positively correlated with the abundance of its endophytic fungi. Similarly, Chen et al. (2021a) reported that the endophytic fungi diversity of *R. palmatum* was positively correlated with the levels of emodin, rhein, and physcion, but negatively connected with the levels of aloe-emodin and chrysophanol. In the case of *Eucommia* bark, its primary active components are closely linked to the presence of endophytic fungi, especially strains from the genera *Phomopsis* and *Diaporthe*, which significantly influence the biosynthesis of bioactive compounds (Liang et al., 2014). Furthermore, another study revealed that the contents of pinoresinol diglucoside and chlorogenic acid in *E. ulmoides* bark showed varying positive correlations with endophytic fungi from an unidentified genus of Davidiellaceae, as well as from *Mortierella*, *Chaetomium*, and *Pestalotiopsis* (Yang et al., 2019). Conversely, *Colletotrichum Corda* and *Penicillium* sp. are significantly negatively correlated with polysaccharide content in *P. Rhizoma*. Similarly, an unclassified fungal taxon demonstrates a strong inverse relationship with polyphenolic compounds. In contrast, *Fusarium* species display a highly significant positive association with 5-hydroxymethylfurfural (Cai et al., 2021). Further supporting these findings, Fan et al. (2021) propose that the dominant endophytic fungal genera in the rhizomes of *P. rhizoma*—including *Setophoma* and *Neocosmospora*—may be likely participate in active metabolite biosynthesis. Notably, endophytic fungal communities within a single plant species exhibit differential regulation of identical bioactive compound biosynthesis and accumulation in traditional Chinese medicinal plants. The endophytic fungus *Trichoderma* sp. in the tubers of *C. yanhusuo* exhibits a positive correlation with the content of tetrahydropalmatine, whereas *Fusarium equiseti*, *Monilochaetes melastomae*, *Clonostachys rosea*, *Mucor racemosus*, and *Fusarium proliferatum* show negative correlations with tetrahydropalmatine levels (Yang et al., 2020). Another study investigated the correlation between endophytic fungi in *S. miltiorrhiza* and the levels of its active compounds. The findings revealed that endophytic fungi of *Torula*, *Cephalosprium*, and *Penicillium* promote the synthesis of salvianolic acid B, whereas *Fusarium* inhibit its synthesis. *Varicospora* facilitate the synthesis and accumulation of salvianic acid A but impede the synthesis and accumulation of tanshinone II_A_ (Wang et al., 2013). Finally, Ma et al. (2015) discovered that the endophytic fungi in *Gynostemma pentaphyllum (G. pentaphyllum)* exhibit significant diversity in both abundance and species composition, and different fungal populations are closely associated with the quality of the plant. Specifically, *Fusarium*, *Cylindrosporium*, and *Leptosphaeria* promote the synthesis and accumulation of Gypenoside A, whereas *Penicillium* inhibit this process. The above content summarizes the correlation between endophytic fungi and the accumulation of active components from Qin medicinal plants. These findings indicate that the abundance and community composition of endophytic fungi are significantly positively or negatively correlated with the content of secondary metabolites (such as alkaloids, saponins, and phenolic acids) in Qin medicinal plants. For example, common endophytic fungi like Fusarium, Penicillium, and Diaporthe can either promote or inhibit the synthesis of specific components. These results reveal the important role of endophytic fungi in shaping the quality of Qin medicinal plants, providing a theoretical basis for improving the quality of medicinal materials by regulating microbial communities.

### Signal regulation between endophytic fungi and Qin medicinal plants

Current research on the molecular mechanisms by which endophytic fungi regulate secondary metabolite biosynthesis in plant hosts remains limited. Endophytic fungi can stimulate host plants by inducing changes in intracellular signaling molecules, activating the expression of defense-related genes, and modulating metabolic pathways. Research has established that *Catharanthus roseus*-associated endophytes upregulate vindoline biosynthesis through transcriptional regulation of key structural and regulatory genes in the terpenoid indole alkaloid (TIA) pathway. In endophyte-inoculated plants, key TIA pathway genes—including geraniol 10-hydroxylase (G10H), tryptophan decarboxylase (TDC), and the transcriptional activator ORCA3 (octadecanoid-responsive *Catharanthus* AP2-domain protein)—were upregulated, while expression of the zinc finger transcriptional repressor ZCT was downregulated. Additionally, vacuolar class III peroxidase (PRX1), responsible for coupling catharanthine and vindoline precursors, showed increased expression. These findings demonstrate that endophytic fungi boost vindoline biosynthesis through coordinated regulation of its key biosynthetic genes (Pandey et al., 2016). Moreover, regulating cellular signal transduction pathways, and altering the expression levels of host cell genes (Lin, 2016).

Li (2017) investigated the effects of *Bupleurum scorzonerifolium* Willd. on the upstream signal transduction pathways in endophytic fungi and its regulatory influence on the expression of key enzyme genes involved in the downstream triterpene saponin biosynthesis pathway. The results demonstrated that *Bupleurum scorzonerifolium* Willd. positively regulates all key enzyme genes associated with triterpene saponin synthesis in endophytic fungi. This finding further explains why the biosynthetic capacity of medicinal plant-derived endophytes to generate host-equivalent bioactive compounds diminishes or even ceases after three-dimensional culture (Li, 2017).

Additionally, Cheng et al. (2022b), Cheng et al. (2024) discovered that the endophytic fungus CHS3 of *Bupleurum scorzonerifolium* Willd. not only produces saikosaponin D but also promotes the accumulation of this bioactive compound in plants. Specifically, the fungus upregulates the expression of key genes (ANT, cypd, CaM, AMPK, AATC, HMGS, HMGR, MVK, MVD, SS, and SE) in plant cells and modulates the MVA pathway, calcium signaling pathway, and AMPK signaling pathway to enhance secondary metabolite biosynthesis (Cheng et al., 2022b; Cheng et al., 2024; Liu et al., 2024).

### Application of multi-omics approaches in the interaction between Qin medicinal plants and endophytes

The interaction between plants and endophytic fungi represents an exceptionally complex biological process. In recent years, multi-omics technologies have provided powerful tools for systematically deciphering this plant-microbe symbiotic system. Integrating genomics, transcriptomics, and metabolomics data enables the construction of a complete causal pathway from genes to transcripts and ultimately to metabolites (Li et al., 2022). For instance, in studying the interaction between rhubarb (*Rheum palmatum*) and its anthraquinone-producing endophytic fungus, integrated multi-omics analysis revealed: the identification of anthraquinone biosynthetic gene clusters in the fungal genome (Wang et al., 2024); observed high expression levels of these gene clusters in co-culture transcriptomes (Chen et al., 2021c); Multi-omics approaches not only hold the potential to decipher the intrinsic mechanisms of plant-endophyte interactions but also establish a solid theoretical foundation for artificially modulating their symbiotic relationships. In the future, this technology is expected to bring revolutionary changes to the cultivation of Qin medicinal plants and even a broader range of crops. By leveraging these strategies, we can enhance plant biomass, optimize the content of high-value compounds, and accelerate the discovery of novel drug lead compounds, thereby paving new pathways for the sustainable utilization of medicinal resources.

## Secondary Metabolites and Bioactivities of Endophytic Fungi Associated with Qin Medicinal Plants

### Endophytic fungi capable of producing active constituents of Qin medicine

**Figure 2:**
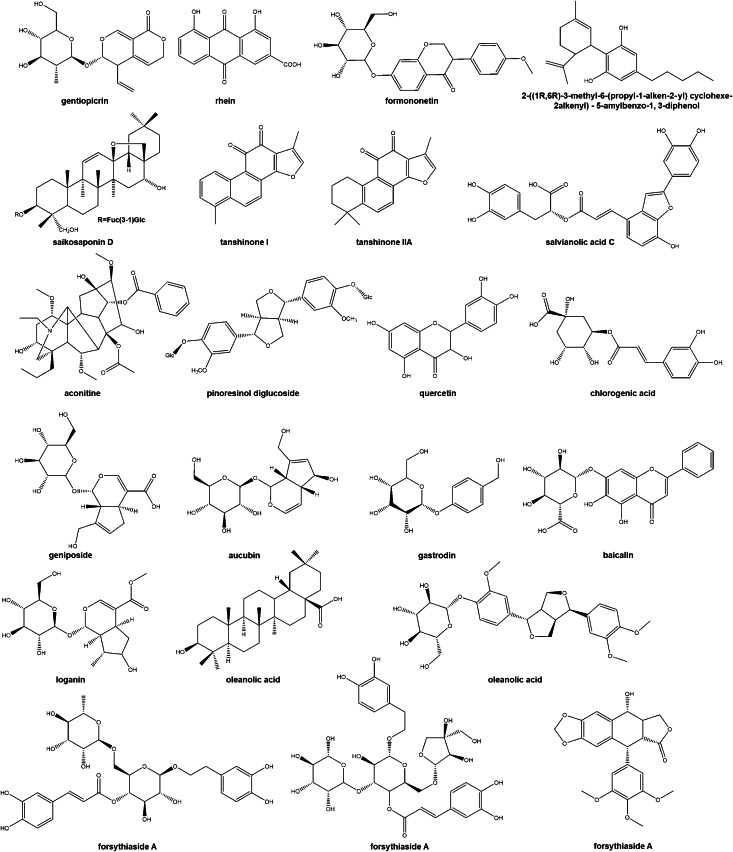
*In vitro* production of bioactive compounds from Qin medicine by endophytic fungi.

With advances in drug discovery, endophytic fungi have become promising alternative producers of plant-derived bioactive compounds. They offer distinct advantages when these metabolites are commercially unavailable, sourced from rare or slow-growing plants, or difficult to synthesize due to structural complexity. Qin medicine primarily refers to authentic medicinal herbs produced in China’s Shaanxi Province and its surrounding regions, which boast a rich variety and are widely utilized in clinical applications due to their diverse active components. Notably, many endophytic fungi associated with Qin medicine have been shown to produce the same bioactive compounds as their host plants (Table 4, Fig. 2). These fungi thus represent potential platforms for the in *vitro* production of Qin medicine’s active constituents.

**Table 4:** Endophytic fungi capable of producing active constituents of Qin medicine.

Qin medicine	Endophytic fungi	Isolation part	Active constituents	Yield	Reference
*Gentiana macrophylla*	/	Root	Gentiopicrin	0.439 mg/L	Yin et al. (2009)
*R. palmatum*	*Fusarium solani* R13	Root	Rhein	5.672 mg/L	You et al. (2013)
*A. Radix*	*Alternaria alternata* HZ13	/	Formononetin	/	Zheng et al. (2017)
	*Coniochaeta ligniaria* HHQ14	Root	2-((1R,6R)-3-methyl-6- (propyl-1-alken-2- yl) cyclohexe-2alkenyl) - 5-amylbenzo-1, 3-diphenol	/	Zhang et al. (2023a)
*Bupleurum chinense*	*Fusarium oxysporum* CHS_2_	Stem	Saikosaponin D	2.17 mg/L	Cheng et al. (2022b)
	*Fusarium acuminatum* CHS3	Stem	Saikosaponin D	2.40 mg/L	Cheng et al. (2022b)
*S. miltiorrhiza*	*Trichoderma atroviride* D16	Root	Tanshinone I	1.119 ± 0.008 μg/g	Ming et al. (2012)
	*Trichoderma atroviride* D16	Root	Tanshinone IIA	3.049 ± 0.001 μg/g	Ming et al. (2012)
	*Phoma glomerata* D14	Leaf	Salvianolic acid C	47.67 ± 0.04 μg/g	Li et al. (2016c)
	*Emericella foeniculicola Udag* TR21	Root	Tanshinones	/	Jiang et al. (2019)
	*Emericella foeniculicola Udag* U104	Root	Tanshinones	/	Jiang et al. (2019)
*Aconitum Lateralis Praeparata*	*Aspergillus flavus* SNGWT-002	Root	Aconitine	52.13 μg/mL	He (2020)
	*Aspergillus parasiticus* SNCWT-003	Seed	Aconitine	56.4 μg/mL	He (2020)
	*Penicillium rubrum* SNWT-014	Stem	Aconitine	12.0 μg/mL	He (2020)
*E. Cortex*	*Phloma* sp.16#	Bark	Pinoresinol diglucoside	13.387 mg/L	Li (2007a)
	*Lateritium sp*.29#	Bark	Pinoresinol diglucoside	4.042 mg/L	Li (2007a)
	*Phloma* sp. 3#	Bark	Pinoresinol diglucoside	0.239 mg/L	Li (2007a)
	*Pythium* sp. 5#	Bark	Pinoresinol diglucoside	0.388 mg/L	Li (2007a)
	*Phloma* sp. 9#	Bark	Pinoresinol diglucoside	0.546 mg/L	Li (2007a)
	*Phloma* sp. 12#	Bark	Pinoresinol diglucoside	0.605 mg/L	Li (2007a)
	*Oospora* sp. 19#	Bark	Pinoresinol diglucoside	0.445 mg/L	Li (2007a)
	*Tolyposporium* sp. 26#	Bark	Pinoresinol diglucoside	0.307 mg/L	Li (2007a)
	*Phomopsis* sp. XP-8	Bark	Pinoresinol diglucoside	11.65 mg/L	Liu et al. (2011a); Shi et al. (2012)
	XP-3	Bark	Pinoresinol diglucoside	0.387 mg/L	Liu et al. (2011a); Shi et al. (2012)
	XP-11	Bark	Pinoresinol diglucoside	3.254 mg/L	Liu et al. (2011a); Shi et al. (2012)
	*Phomopsis* sp. SX2018-06	Bark	Pinoresinol diglucoside	43.28 mg/L	Cheng et al. (2020)
	*Chaetophoma Cke* DZ11	Stem	Quercetin	0.418 mg/L	Shen (2008)
	*Sordariomycete* sp. B5	Stem	Chlorogenic acid and geniposide	/	Chen et al. (2010)
	*Alternaria* sp. 9/11/16/26/30	Leaves and fruits	Aucubin	0.685–1.114 mg/mL	Yan (2015)
	*Cladosporium* sp. 31/49	Leaves and fruits	Aucubin	0.258/0.572 mg/mL	Yan (2015)
	*Niqrospora* sp.4	Leaves and fruits	Chlorogenic acid	0.12 mg/mL	Yan (2015)
	*Alternaria* sp.7	Leaves and fruits	Chlorogenic acid	0.114 mg/mL	Yan (2015)
*G. elata*	*Chaetomium* sp. DF101	Tuber	Gastrodin	0.057 mg/g	Mo (2009)
	*Fusarium* sp. BY302	Tuber	Gastrodin	0.089 mg/g	Mo (2009)
	*Armillaria* sp. SC108	Tuber	Gastrodin	0.184 mg/g	Mo (2009)
	*Oidiodendron* sp. GeR6	Tuber	Gastrodin	39.2 μg/g	Liang et al. (2020)
*S. baicalensis*	*Penicillium* sp. J1	Stem	Baicalin	/	Wei et al. (2012)
	*Penicillium* sp. H3	Flower	Baicalin	/	Wei et al. (2012)
*Cornus officinalis*	*Alternaria eichhorniae* G9	Fruits	Loganin	62 μg/L	Gao (2019)
*R. Polygalae*	*Alternaria Nees* YZ-31	Root	Oleanolic acid	/	Fu & Xiong (2012)
*G. pentaphyllum*	*Helotiales* G4	Root	Gypenoside	69 μg/L	Shang et al. (2021); Shang et al. (2011)
*Forsythia suspensa*	*Colletotrichum gloeosporioides* G10	fruits	Phillyrin	56 μg/L	Zhang (2013); Zhang, Wei & Wang (2012)
	*Cladosporium* sp. Y2	Leaves	Phillyrin	12.6941 μg/mL	Niu et al. (2018)
			Forsythiaside A	1.2123 μg/mL	Niu et al. (2018)
			Forsythiaside B	0.7512 μg/mL	Niu et al. (2018)
	*Cladosporium* sp. H2	Flower	Forsythiaside A	3.2543 μg/mL	Niu et al. (2018)
	*Cladosporium* sp. H1	Flower	Forsythiaside B	0.6977 μg/mL	Niu et al. (2018)
	*Aspergillus* sp. J1	Stem	Phillyrin	32.5587 μg/mL	Niu et al. (2018)
			Forsythiaside A	0.5595 μg/mL	Niu et al. (2018)
*S. hexandrum*	*Monilia* sp. 97T22	Subterranean stem	Podophyllotoxin	/	Li, Zeng & Zhang (1999)
	*Alternarian Nees* ex Wall. Ty	/	Podophyllotoxin	2.418 μg/L	Zhang (2006)
	*Mucormicheliex Fries* Tc/Td/Te/Mi	Subterranean stem	Podophyllotoxin	/	Zhang et al. (2008)
	*Cephalosporium* sp. T8	/	Podophyllotoxin	/	Liu et al. (2011b)
	*Mucor fragilis Fresen*. TW5	Subterranean stem	Podophyllotoxin	49.3 mg/g	Huang et al. (2014)
	*Fusarium* sp. TG9	root	Podophyllotoxin	132.83 mg/mL	Liang et al. (2016)
	*Pleosporales* sp. MF565 (G-1)	root	Podophyllotoxin	8.759 μg/L	Wang et al. (2017b)
	*Pseudallescheria* sp. T55 (G-2)	root	Podophyllotoxin	30.51 μg/L	Wang et al. (2017b)
	*Hypocreales* sp. MF489 (G-3)	root	Podophyllotoxin	6.664 μg/L	Wang et al. (2017b)
	*Hypocreales* sp. LF213 (G-4)	root	Podophyllotoxin	13.45 μg/L	Wang et al. (2017b)
	*Cyphellophorareptans strain* CBS (G-5)	root	Podophyllotoxin	13.182 μg/L	Wang et al. (2017b)
	*Chaetomium globosum* strain MF564 (J-1)	Stem	Podophyllotoxin	/	Wang et al. (2017a)
	C. sp. 4RF3 (J-2)	Stem	Podophyllotoxin	/	Wang et al. (2017a)
	*Pseudallescheria* sp. T55 (J-3)	Stem	Podophyllotoxin	/	Wang et al. (2017a)

### Endophytic fungi produce novel secondary metabolites with distinctive structural features

Endophytic fungi in plants have emerged also as a significant source of novel natural products. To date, a wide array of structurally diverse compounds, including alkaloids, steroids, terpenoids, and others Fig. S1, have been isolated and characterized from Qin medicinal plant-associated endophytic fungi. These compounds have substantially enriched the repertoire of natural products. The secondary metabolite Salchaeto globosin B (10), isolated and identified from the endophytic fungus *Chaetomium globosum* D38 (*C. globosum* D38) in *S. miltiorrhiza* roots, exhibits moderate inhibitory effects against colon cancer cells (HCT-116) and prostate cancer cells (PC-3) (Ji, 2020). Table 5 provides a summary of the novel compounds produced by endophytic fungi of Qin medicine.

**Table 5:** Novel secondary metabolites produced by endophytic fungi of Qin medicinal plants.

Novel compounds	Type	Endophytic fungi	Plants	Reference
Subplenones A-J	Dimeric Xanthones	*Subplenodomus* sp. CPCC 401465	*Gentiana straminea* Maxim.	Cai et al. (2023)
Cochlioquinone B1	acetylcholine colloidone	*Bipolaris sorokiniana* KLBMPSM007	*S. miltiorrhiza*	Ding (2017)
Salchaetoglobosin A-B	Indole alkaloids	*C. globosum* D38	*S. miltiorrhiza*	Ji (2020)
Pinophicin A	Terpene	*Talaromyces pinophilus*	*S. miltiorrhiza*	Zhao (2020)
Pinophol A	Polyene	*Talaromyces pinophilus*	*S. miltiorrhiza*	Zhao (2020)
(7S, 8S, 11R)- aureonitol A-B	Ketones	*Chaetomium globosum*	*S. miltiorrhiza*	Zhao (2020)
Colletotricholide A-B	Terpenoids	*Colletotrichum gloeosporioides*	*S. miltiorrhiza*	Zhao (2020)
(14β,22E)-9, 14-dihydroxyergosta- 4,7,22-triene-3,6-dione	Steroids	*Phomopsis* sp.	*Aconitum carmichaeli* Debx	Wu et al. (2013)
(5α,6β,15β,22E)- 6-ethoxy-5,15- dihydroxyergosta- 7,22-dien-3-one	Steroids	*Phomopsis* sp.	*Aconitum carmichaeli* Debx	Wu et al. (2013)
6-O-demethyl-4- dehydroxyaltersolanol A	Anthraquinones	*Nigrospora* sp. YE3033	*Aconitum carmichaeli*	Zhang et al. (2016)
8,11- didehydrochermesinone B	Azaphilones	*Nigrospora* sp. YE3033	*Aconitum carmichaeli*	Zhang et al. (2016)
(7S)-7-hydroxy-3,7- dimethylisochromene-6,8- dione	Azaphilones	*Nigrospora* sp. YE3033	*Aconitum carmichaeli*	Zhang et al. (2016)
Shikimerans B-C	Polyketides	*Chaetomium* sp. FZ-4	*Aconitum carmichaelii* Debx	Chen et al. (2021e)
Penicichromanone A-B	Chromone	*Penicillium Chrysogenum*	*E. Cortex*	Liu (2020)
14-hydrocyclopeptin	Dehydrocyclopeptin	Penicillium sp. GZWMJZ-042	*E. Cortex*	Wang et al. (2023b)
Epiterenoid A-C	Polyoxygenated meroterpenoids	*Epicoccum* sp. YUD17002	*G. elata*	Li et al. (2019)
Armiloid A	Illudalane derivative	*Epicoccum* sp. YUD17002	*G. elata*	Li et al. (2019)
Epicoterpene A-E	Protoilludane-type sesquiterpenes	*Epicoccum* sp. YUD17002	*G. elata*	Li et al. (2020)
Armilliphatic A-C	Aryl esters	*Epicoccum* sp. YUD17002	*G. elata*	Li et al. (2020)
Phometides A-B	Polyketide	*Phoma* sp. YUD17001	*G. elata*	Yang et al. (2024b)
Terrustone	Trihydroxycyclopentanone	*Aspergillus terreus* HQ100X-1	*Scutellaria formosana*	Huang et al. (2022)
Asperteretone G	Butyrolactone	*Aspergillus terreus* HQ100X-1	*Scutellaria formosana*	Huang et al. (2022)
8Z-isomer of lucilactaene	Lucilactaene	*Fusarium* sp. QF001	*S. baicalensis*	Maharjan et al. (2020)
4Z-isomer of lucilactaene	Lucilactaene	*Fusarium* sp. QF001	*S. baicalensis*	Maharjan et al. (2020)
(-)-4,6′- anhydrooxysporidinone	tricyclic pyridone alkaloids	*Fusarium lateritium* SSF2	*Cornus officinalis*	Choi & Shim (2017)
(-)-6-deoxyoxysporidinoCne	tricyclic pyridone alkaloids	*Fusarium lateritium* SSF2	*Cornus officinalis*	Choi & Shim (2017)
(-) sambutoxin	tricyclic pyridone alkaloids	*Fusarium lateritium* SSF2	*Cornus officinalis*	Choi & Shim (2017)
Bipolacochlioquinones A-C	Meroterpenoids	*Bipolaris victoriae* S27	*Rubia podantha Diels*	Feng et al. (2022)
Bipoterprides A-L	Terpene-nonadride heterodimers	*Bipolaris victoriae* S27	*Rubia cordifolia*	Feng & Cui (2024)
Bipolodrides A-B	Terpene monomers	*Bipolaris victoriae* S27	*Rubia cordifolia*	Feng & Cui (2024)
Bipolenin O	Nonadride monomers	*Bipolaris victoriae* S27	*Rubia cordifolia*	Feng & Cui (2024)
Pestalotiopsins D-G	Caryophyllene-type sesquiterpenes	*Pestalotiopsis adusta*	*S. hexandrum (Royle) Ying*	Xiao et al. (2017)
Pestalotiophol A-B	Drimane-type sesquiterpenes	*Pestalotiopsis adusta*	*S. hexandrum (Royle) Ying*	Xiao et al. (2017)

### Bioactive secondary metabolites from endophytic fungi associated with Qin medicinal plants

#### Antibacterial bioactive secondary metabolites

Endophytic fungi exhibit significant possible as antimicrobial agents. A study has shown that the secondary metabolite azaphilone alkaloids and 24 other bioactive compounds produced by the endophytic fungus *Cochliobolus* sp. APS1 (isolate code) derived from *Andrographis paniculata* demonstrates potent antimicrobial efficacy against ten clinically relevant pathogens, with pronounced activity against methicillin-resistant *Staphylococcus aureus* (MRSA) and vancomycin-resistant *S. aureus* (VRSA). The thermostable, non-proteinaceous antimicrobial components disrupt bacterial viability through dual mechanisms: (i) inhibition of key glycolytic enzymes, and (ii) induction of membrane integrity loss evidenced by extracellular leakage of K^+^ ions and biomacromolecules (DNA/proteins). The biomolecules derived from APS1 can inhibit biofilm formation in bacterial pathogens and block essential acetylcholinesterase, thereby achieving antibacterial effects (Santra, Maity & Banerjee, 2022).

Studies have demonstrated that endophytic fungi derived from Qin medicinal plants possess diverse antibacterial activities, particularly strains isolated from the following medicinal species: *Gentiana macrophylla* (*G. macrophylla*) leaves (Li et al., 2022), *A. Radix* (Cui et al., 2007; Li et al., 2010; Zhou et al., 2012a; Zhou et al., 2012b), *Bupleurum chinense* (Li, Luo & Zhang, 2015), *S. miltiorrhiza* (Wang et al., 2021a), *Aconitum carmichaeli Debx* (He, 2020; Li et al., 2009; Xia et al., 2019), *Cornus officinalis* (Gao, 2019; Zhao et al., 2022; Zhao et al., 2020) and *E. cortex* (Li, 2007a; Li, 2007b; Ma, Tian & Zhang, 2011; Sun, 2005; Yang et al., 2012a; Yang et al., 2012b; Zhu, 2005). For example, differanisole A, 2,6-dichloro-4-propylphenol, and 4,5-dimethylresorcinol, isolated from the endophytic fungus *Chaetomium* sp. HQ-1 in the leaves of *Astragalus chinensis*, exhibit moderate antibacterial activity against *S. aureus, methicillin-resistant S. aureus (MRSA),* and *Listeria monocytogenes* (*L. monocytogenes*) (Liu et al., 2019). In addition to exhibiting activity against human pathogenic microorganisms, multiple compounds with potent anti-agricultural pathogen activity have been isolated and identified from the secondary metabolites of various endophytic fungi associated with Qin medicinal plants. These compounds demonstrate significant potential for application in biocontrol applications against phytopathogens. Table 6 summarizes the antimicrobial compounds isolated and identified from secondary metabolites of endophytic fungi in Qin medicinal plants, along with their MIC and IC_50_ values against target organisms.

**Table 6:** Antimicrobial active secondary metabolites.

Compound	Activity (target organism)	MIC/ IC_50_	Source fungus	Host plant	Reference
differanisole A, 2,6-dichloro-4-propylphenol and 4,5-dimethylresorcinol	Antibacteria l (*S. aureus, methicillin-resistant S. aureus (MRSA),* and *L. monocytogenes*)	*/*	*Chaetomium* sp. HQ-1	*Astragalus chinensis*	Liu et al. (2019)
isochromophilone IV, epi-isochromophilone III	Bacteriostasis (*Candida glabrata* and *S. aureus*)	16–32 μg/mL	GZWMJZ-068	*E. Cortex*	Gong et al. (2018)
cyclo (Pro-Ile) dipeptide	*E. coli*	9.83 mg/mL	*Alternaria* sp	*E. Cortex*	Wu (2018)
cyclo-(Phe-hydroxypro) dipeptide	*S. aureus* and *E. coli*	9.50 mg/mL and 9.50 mg/mL	*Alternaria* sp	*E. Cortex*	Wu (2018)
(R)-7-hydroxyaspergillin	antifungal	/	*Trichoderma* sp. FZ-31	*Aconitum carmichaelii*	Chen et al. (2021d)
indole-3-acetic acid and trichothecin A	antibacterial	/	*Trichoderma* sp. FZ-31	*Aconitum carmichaelii*	Chen et al. (2021d).
gliotoxin and dehydrogliotoxin	antibacterial and antifungal	/	ER16-5	*E. Cortex*	Zhang & An (2019)
phometide A and Phometide B	*S. aureus*	4 μg/mL and 16 μg/mL	*Phoma* sp. YUD17001	*G. elata*	Yang et al. (2024b)
5-hydroxymethylfuran- 3-carboxylic acid	*Fusarium oxysporum f. sp*	6.25 µg /mL	EL09	*E. Cortex*	Yang et al. (2012b)
griseofulvin	*Botrytis cinerea, Colletotrichum orbiculare, Didymella bryoniae* and *Sclerotinia sclerotiorum*	0.68, 0.38, 0.91 and 0.61 mg/L	zjqy610	*P. Rhizoma*	Wang et al. (2010)
cyclo-(Tyr-Pro) and cyclo-(Phe-hydroxypro)	*Alternaria sp*	10.31 mg/mL and 4.97 mg/mL	*Alternaria* sp.	*E. Cortex*	Wu (2018)

#### Antitumor secondary metabolites with potent antiproliferative activity

The extraction of anti-cancer active compounds from plants faces several challenges, including low compound concentrations and the endangered status of plant resources. The application of medicinal plant-associated endophytic fungi represents a viable strategy to overcome these challenges. To date, multiple endophytic fungi derived from Qin medicine have been confirmed to exhibit significant anti-tumor activity. For instance, Cheng et al. (2016) found that the fermentation broth of *Alternaria alternata* CHS4, an endophytic fungus isolated from *Bupleurum scorzonerifolium* Willd, exhibited an inhibitory effect on liver cancer cells HepG-2 that was positively correlated with its concentration, and its half-maximal inhibitory concentration (IC_50_) was 75.11 μg/mL. Endophytic fungi (*n* = 122) isolated from *S. miltiorrhiza* were screened using HepG2 cells *in vitro*, revealing that *Bipolaris sorokiniana* KLBMPSM007 exhibited measurable anti-proliferative effects in this model system (Ding, 2017). Wang et al. (2023a) obtained a novel compound (14-hydrocyclopeptin) along with four known metabolites through 5-azacytidine-induced fermentation of *Penicillium* sp. GZWMJZ-042, an endophytic fungus isolated from *E. cortex* leaves. Preliminary screening showed these compounds exhibited *α*-glucosidase inhibitory potential with in *vitro*. These results collectively suggest that Qin medicinal plants endophytic fungi represent a valuable source for discovering potentially applicable antitumor compounds. Table 7 summarizes the antitumor-active secondary metabolites isolated from various Qin medicinal endophytic fungi, including their structural characteristics and IC_50_ values against target organisms.

**Table 7:** Antitumor-active secondary metabolites.

Compound	Activity (target organism)	IC_50_	Source fungus	host plant	Reference
Lupinine	Adenosine deaminase inhibition	21.42 mM	*Aspergillus niger* QJ-4	*G. macrophylla*	Zhang et al. (2018b)
3-(4-Nitrophenyl)- 5-phenylisoxazole	Cytotoxicity (HepG2)	0.347 mM	*Aspergillus niger* QJ-4	*G. macrophylla*	Zhang et al. (2018b)
	Cytotoxicity (SMCC-7721, )	0.380 mM	*Aspergillus niger* QJ-4	*G. macrophylla*	Zhang et al. (2018b)
Kaempferol-3-O- β-diglucoside	Adenosine deaminase inhibition	6.13 mM	*Aspergillus niger* QJ-4	*G. macrophylla*	Zhang et al. (2018b)
Myricetin	Adenosine deaminase inhibition	8.19 mM	*Aspergillus niger* QJ-4	*G. macrophylla*	Zhang et al. (2018b)
Cyclo-(Pro-Gly), Cyclo-(Pro-Leu), Cyclo-(Pro-Val)	Cytotoxicity (multiple tumor cell lines)	/	*Aspergillus* sp. AR-17-2	*Astragalus membranaceus*	Liu et al. (2020)
Salchaetoglobosin B	HCT-116 (colon cancer)	60.42 μM	*C. globosum* D38	*S. miltiorrhiza*	Ji (2020)
	PC-3 (prostate cancer)	62.21 μM	*C. globosum* D38	*S. miltiorrhiza*	Ji (2020)
Pestalotiopsin C	SMMC-7721 (liver cancer)	Comparable to etoposide	*Pestalotiopsis* sp.	*S. hexandrum*	Xiao et al. (2017)
Pestalotiopsin A	A549, HeLa, SMMC-7721	Comparable to etoposide	*Pestalotiopsis* sp.	*S. hexandrum*	Xiao et al. (2017)
14-hydrocyclopeptin	α-Glucosidase inhibition	147.0 μg/mL	*Penicillium* sp. GZWMJZ-042	*E. Cortex*	Wang et al. (2023b)
Viridicatol	α-Glucosidase inhibition	118.7 μg/mL	*Penicillium* sp. GZWMJZ-042	*E. Cortex*	Wang et al. (2023b)
4a-demethylpaspaline- 4a-carboxylic acid	α-Glucosidase inhibition	110.5 μg/mL	*Penicillium* sp. GZWMJZ-042	*E. Cortex*	Wang et al. (2023b)
(-)-Chaetominine	α-Glucosidase inhibition	65.9 μg/mL	*Penicillium* sp. GZWMJZ-042	*E. Cortex*	Wang et al. (2023b)
Cyclo(dehydrohistidyl- L-tryptophyl)	α-Glucosidase inhibition	74.6 μg/mL	*Penicillium* sp. GZWMJZ-042	*E. Cortex*	Wang et al. (2023b)
Dankasterones A	α-Glucosidase inhibition	40.6 μg/mL	*Penicillium* sp. GZWMJZ-042	*E. Cortex*	Wang et al. (2023b)
2′-deoxythymidine	α-Glucosidase inhibition	151.5 μg/mL	*Penicillium* sp. GZWMJZ-042	*E. Cortex*	Wang et al. (2023b)
Armilliphatic A	HL-60 (leukemia)	15.80 μM	*Epicoccum sp*. YUD17002 (co-cultured with *Armillaria* sp.)	*G. elata*	Li et al. (2020)
	A-549 (lung cancer)	15.93 μM			
	SMMC-7721 (liver cancer)	19.42 μM	*Epicoccum* sp. YUD17002	*G. elata*	Li et al. (2020)
	MCF-7 (breast cancer)	19.22 μM	*Epicoccum* sp. YUD17002	*G. elata*	Li et al. (2020)
	SW480 (colon cancer)	23.03 μM	*Epicoccum* sp. YUD17002	*G. elata*	Li et al. (2020)

#### Secondary metabolites exhibiting antioxidant activity

The secondary metabolites of multiple endophytic fungi associated with Qin medicinal plants have been confirmed to exhibit significant antioxidant activity. For example, Cheng et al. (2022a) isolated 78 endophytic fungi from *Gentiana straminea* Maxim. and identified one strain, *Cadophora* sp. GS-6, capable of producing isoorientin (1.569 mg/L), quercetin (1.110 mg/L), and isovitexin (0.824 mg/L). The extract of this strain demonstrated a DPPH radical scavenging activity with an IC_50_ value of 39.9 μg/mL.

Several bioactive components with significant antioxidant properties have been derived from the secondary metabolites of endophytic fungi associated with Qin medicinal plants. For example, Liang et al. (2020) found that the secondary metabolites of two endophytic fungi, *Oidiodendron* GeR6 and *Ascomycota* sp. GeR2, isolated from *G. elata* tubers exhibited notable antioxidant activity, with IC_50_ values of 456.42 μg/mL and 303.84 μg/mL, respectively. The effective components were likely flavonoids, present at concentrations of 9.22 ± 0.09 mg/g and 9.03 ± 0.13 mg/g, respectively.

Wang (2020) screened 28 endophytic fungi from *S. miltiorrhiza* roots and identified an endophytic *Fusarium* HBG16 strain with both antioxidant and anticoagulant activities. The mycelial polysaccharides of this strain demonstrated a DPPH radical scavenging rate of 53.6% at a concentration of one mg/mL. Huo & Chen (2004) found that the extract of *Chaetomella* sp., an endophytic fungus from *E. cortex* leaves, exhibited significant antioxidant activity. Xie & Chen (2009) reported that polyphenols were the primary antioxidant compounds in the metabolites of another endophytic fungus from *E. cortex* leaves, with a content of 255.53 ± 1.38 mg/g. Wu (2018) isolated and identified 11 monomer compounds from the fermentation products of *Alternaria* sp., an endophytic fungus from *E. cortex* leaves, all of which showed varying degrees of DPPH or hydroxyl radical scavenging activity.

#### Secondary metabolites exhibiting additional biological activities

Zhai (2019) isolated and purified the polysaccharides from the endophytic fungi that produce tanshinone and obtained the homogeneous polysaccharide PSF-W-1 with the highest content. This polysaccharide is composed of mannose, glucose and galactose and is a heteropolysaccharide. Research shows that PSF-W-1 is the main active component that promotes the growth of *S. miltiorrhiza* plants and the accumulation of tanshinones. Zhang et al. systematically studied the extracellular polysaccharides in the fermentation broth of the endophytic fungus JY29 of *G. pentaphyllum*. Under optimized fermentation conditions, the yield can reach 271.40 mg/L. Further experiments showed that when trace amounts of gypenosides and puerarin were added, the yields of extracellular polysaccharides increased to 3.88 g/L and 3.67 g/L, respectively. The study also confirmed that this extracellular polysaccharide has multiple biological activities such as good antioxidant, antibacterial, anticancer, anti-inflammatory and immune-enhancing properties. In addition, this polysaccharide is expected to be used as a biological feed to enhance the immunity and promote the growth and development of broilers (Fu, 2022; Jiao, 2016; Li, 2016; Li et al., 2016a; Wang et al., 2019).

### Application of multi-omics in the discovery of novel metabolites from medicinal plants in Qin medicine

Multicomics technologies have demonstrated powerful capabilities in systematically analyzing novel metabolites of Qin medicinal plants. By integrating multidimensional data from genomics, transcriptomics, and metabolomics, this approach comprehensively reveals previously unrecognized active components and their biosynthetic mechanisms. For instance, mass spectrometry-based untargeted metabolomics (*e.g.*, UPLC-MS/MS) has been successfully applied to species such as *E. cortex* and Gentiana macrophylla, leading to the systematic identification of numerous metabolites. Notably, 42 new compounds were first discovered in Gentiana macrophylla, substantially expanding the known chemical diversity of Qin medicine (Chen & Zeng, 2018). In elucidating the origins of Dao-di herbs, integrated analysis combining comparative genomics and metabolomics has shown that the accumulation of specific terpenoid components (such as gentiopicroside) in the genuine producing areas of Gentiana macrophylla is not only regulated by genetic background but also closely related to environmental factors such as precipitation (Liang, 2022). In the future, multi-omics approaches will move beyond conventional methods to allow for the systematic mining of novel plant metabolites. By providing deep insights into metabolic networks, they will drive the rapid discovery of drug leads with new structures and distinct activities.

## The Potential Application of Endophytic Fungi Associated with Qin Medicinal Plants

### Enhance the stress tolerance of plants

#### Enhance the disease resistance of plants

Endophytic fungi serve as natural biocontrol agents, enhancing plant disease resistance through multiple mechanisms and offering a novel solution for sustainable agriculture. The improper and extreme use of pesticides has led to the growth of antibiotic resistance in plant pathogenic microorganisms, thereby facilitating the evolution of drug-resistant fungal pathogens. Chemical fungicides are not only economically burdensome but also have substantial adverse environmental impacts. Leveraging endophytic fungi as biocontrol agents can effectively mitigate plant diseases and promote sustainable agricultural practices. Endophytic fungi protect their host plants from pathogen invasion throughout their life cycle *via* mechanisms such as antibiotic production, parasitism, and competition. Multiple research has demonstrated that inoculating endophytic fungi associated with Qin medicinal plants onto their host plants can significantly enhance their disease resistance. Inoculating specific Qin medicine endophytic fungi into other crops can also effectively reduce the incidence of plant diseases.

**(1) Disease resistance applications of rhubarb endophytic fungi**

Chen et al. (2022a) found that the fermentation broth of the endophytic fungus *Trichoderma citrinoviride* HT-1 isolated from the roots of *R. palmatum* could significantly increase the activity of plant-induced defense enzymes and achieve a disease control rate of 72.53% against root rot of *R. palmatum*.

**(2) Disease resistance applications of** ***G. elata*** **endophytic fungi**

Additionally, Liu (2019) isolated the endophytic fungus *Acremonium* JY.TM-6 from the tubers of *G. elata* and co-inoculated it with *Candida vartiovaarae* of *G. elata*, *Pseudomonas* of *G. elata*, *Penicillium oxalicum* of *G. elata*, *A. membranaceus* and *M. officinalis* onto healthy *G. elata*. The inhibition rates of JY.TM-6 against these five pathogenic fungi were 16.67%, 66.67%, 33.33%, 50.00%, and 33.33%, respectively.

**(3) Cross-crop disease resistance applications of** ***E. cortex*** **endophytic fungi**

The endophytic fungus *Thielavia* DZGS08, from *E. cortex*, shows strong inhibitory effects on maize *Rhizoctonia solani* (Ding et al., 2014; Sun & Ding, 2013). Ding et al. (2015) further investigated the colonization ability and efficacy of DZGS07 in wheat roots. Their results indicated that DZGS07 can tolerate carbendazim and significantly enhance the activities of three defense enzymes—polyphenol oxidase (PPO), peroxidase (POD), and phenylalanine ammonia lyase (PAL)—in wheat roots. Consequently, the incidence of wheat sheath blight was reduced from 90.25% in the control group to 13.36% after treatment with DZGS07, highlighting its potential as an effective agricultural biocontrol agent. Li (2023) found that the endophytic fungus Y232 strain from *E. cortex* exhibited inhibitory effects on multiple plant pathogenic fungi in *vitro*. When applied to potted corn, the fungal suspension significantly suppressed the damage caused by *Setosphaeria turcica* maydis and improved the plant height, fresh weight, dry weight, and stem length of corn seedlings. Collectively, these findings underscore the versatile roles of endophytic fungi from Qin medicinal plants as effective agents for biological disease control across multiple plant systems.

#### Enhance the antioxidant capacity of plants

Antioxidant activity primarily refers to the elimination of reactive oxygen species (ROS) produced by organisms under external stress conditions. Under drought stress, the balance between ROS and their scavenging systems in plant metabolic pathways is disrupted. Endophytic fungi can enhance host survival in adverse environments by increasing the levels of osmoregulatory substances and boosting antioxidant activity. Yang et al. (2009) discovered that the endophytic fungus *Aspergillus flavus* from *S. miltiorrhiza* significantly induces the expression of POD, catalase (CAT), and PPO in *S. miltiorrhiza* suspension cells, demonstrating protective effects against reactive oxygen species (ROS)-mediated damage under stress conditions. Lin (2018) reported that five endophytic fungi in *S. miltiorrhiza* can significantly enhance the activities of SOD, POD, and CAT in *S. miltiorrhiza* test-tube seedlings, thereby improving the plant’s stress resistance. Thus, endophytic fungi can effectively alleviate oxidative stress by activating the host’s antioxidant enzyme system, providing a novel microbial-mediated approach to enhance stress resistance in medicinal plants.

### As an inducer applied in plant tissue culture

#### Inducers promote tanshinone biosynthesis

Inducers can quickly, precisely, and selectively stimulate the expression of particular genes in cultured plant cells, thus increasing the yield of valuable secondary metabolites. Ming et al. (2013) discovered that the endophytic fungus *T. atroviride* D16 strain strainin *S. miltiorrhiza* roots not only produces tanshinone I and tanshinone IIA but also its mycelial extract promotes the growth of hairy roots and stimulates tanshinone biosynthesis. Zhai (2019) research demonstrated that active monosaccharides from D16 strain can specifically bind to receptors on the surface of *S. miltiorrhiza* cells, triggering changes in the upstream signaling molecule H_2_O_2_ andtransmitting signals to downstream molecules NO and ABA. This leads to altered expression levels of the key transcription factors SmERF1B, SmGRF1, and SmMYB86, thereby regulating protein synthesis of enzymes involved in the tanshinone biosynthesis pathway and promoting tanshinone production (Zhai, 2019). Furthermore, Zhai et al. (2017) found that another endophytic fungus, *C. globosum* D38, exhibits lower toxicity compared to D16 strain and significantly increases the content of dihydrotanshinone I and cryptotanshinone in hairy roots (by eight-fold and 14.9-fold, respectively). Liu’s study indicated that unidentifiedco-culturing endophytic fungi TR21, NU251, U104, and NU204 with *S. miltiorrhiza* tissues enhances tanshinone IIA synthesis, while the culture broth of endophytic fungi TR21, NU251, U104, and NU151 increases tanshinone IIA content in callus tissue (Liu, 2015).

#### Inducers regulate flavonoid biosynthesis

Zhang et al. (2023a) and Zhang et al. (2023b) reported that the culture solutions of the endophytic fungi *Alternaria alternate* CL79 and *Fusarium solani* CL105, isolated from *S. baicalensis*, significantly promoted the synthesis of four key flavonoids—baicalin, wogonoside, baicalein, and wogonin—when added as inducers to the callus culture system of *S. baicalensis*. Specifically, after induction with the CL79 culture solution, the contents of these flavonoids increased by 37.8%, 40.4%, 44.7%, and 42.2%, respectively. In contrast, induction with the CL105 culture solution resulted in even more substantial increases: 78.1%, 140.9%, 275.6%, and 208.5%, respectively (Zhang et al., 2023a; Zhang et al., 2023b). The study suggested that the CL105 culture solution may enhance flavonoid synthesis by stimulating the expression of *myb3*, *myb8*, *myb13*, *gaox*, and *cyp707a* genes, leading to significant up-regulation of chalcone synthase (CHS), chalcone isomerase (CHI), flavone synthase (FNS), and flavanone 6-hydroxylase (F6H) genes involved in the flavonoid biosynthesis pathway (Cui et al., 2024). Additionally, Xu et al. (2022) demonstrated that the endophytic fungus *Pseudomonas* sp. 2B, isolated from the root of *S. baicalensis*, could reprogram the defense-related secondary metabolism in *S. baicalensis* Georgi, promoting the accumulation of baicalein, wogonin and chrysin in the callus tissue. These results collectively demonstrate that endophytic fungi serve not only as symbiotic partners but also as potent elicitors of secondary metabolism in medicinal plants.

### As bio-fertilizer to promotes plant growth and enhances the accumulation of secondary metabolites

Endophytic fungi and their bioactive compounds are widely recognized as biostimulants. When co-cultured with host plants, they can induce or stimulate the biosynthesis of specialized metabolites in host plants, thereby improving the quality of medicinal materials. Studies have shown that indole-3-acetic acid (IAA) biosynthesis plays a crucial role in modulating root system development, improving mineral absorption, and strengthening plant tolerance to multiple environmental stressors such as water deficit, salt stress, and heavy metal contamination. The exogenous application of IAA not only stimulates crop growth but also optimizes nutrient utilization, potentially decreasing the reliance on synthetic fertilizers. This strategy presents an eco-friendly solution for advancing sustainable farming practices (Etesami & Glick, 2024). Du et al. (2023) identified two endophytic fungi from 54 strains of *Bupleurum chinense* DC. endophytes that are capable of secreting IAA, with secretion levels of 59.55 mg/L and 76.16 mg/L, respectively. These isolates demonstrate significant potential for application in promoting plant growth. Siderophores can mitigate metal toxicity in plants and enhance phytoremediation efficacy. Notably, in highly heavy metal-contaminated and strongly alkaline soils, siderophore treatment has been shown to effectively stimulate plant growth (Grobelak & Hiller, 2017). Additionally, the crosstalk in plant hormone signaling pathways has been confirmed as an important route through which endophytic fungi influence plant growth and metabolite accumulation. The interdependence of endophyte-mediated phytohormonal modulation and secondary metabolite biosynthesis varies according to both host plant taxonomy and fungal endophyte characteristics.

#### Promoting the growth and metabolite accumulation in *R. palmatum*

Chen et al. (2021a) discovered that the endophytic fungi *Trichoderma atroviride* LX-7 and *Trichoderma citrinoviride* HT-1, isolated from the roots of *R. palmatum*, possess the ability to produce siderophores and IAA, as well as to solubilize organic and inorganic phosphates. Inoculation with these two endophytic fungi significantly enhanced the germination rate of pakchoi seeds and the growth quality of seedlings. Experimental results demonstrated that the optimal effects were observed when the two endophytic fungi were used in combination (Chen et al., 2021a). Furthermore, inoculation of *R. palmatum* seedlings with *Trichoderma citrinoviride* HT-1 significantly encouraged seedling development and metabolite accumulation, with the promoting effects mainly related to upregulated expression of genes associated with plant hormone signal transduction, secondary metabolism, plant-pathogen interactions, and phenylpropanoid biosynthesis pathways. QRT-PCR analysis of selected differentially expressed genes shown that genes connected to plant hormone transduction Gretchen Hagen 3 (GH3) and ethylene response factor (ERF)) were upregulated by 2.59-fold and 2.71-fold, respectively; three genes involved in secondary metabolite biosynthesis aloe ketone synthase (ALS), CHS, and benzylacetone synthase (BAS)) showed 4.31-fold, 2.97-fold, and 2.68-fold upregulation, respectively; while two genes associated to plant-pathogen interactions and phenylpropanoid biosynthesis HSP and POD exhibited 4.13-fold and 4.08-fold upregulation compared to kinase controls (Chen et al., 2022b). Chen et al. (2022b) demonstrated that endophytic fungi derived from *R. palmatum* enhance both host growth and secondary metabolite accumulation by secreting growth-promoting substances and upregulating key genes in hormone signaling and phenylpropanoid biosynthesis pathways.

#### Promoting the growth and metabolite accumulation in *S. miltiorrhiza*

Tang, Li & Guo (2014) inoculated the endophytic fungus *Paecilomyces* sp. SMF34, isolated from the stems of *S. miltiorrhiza*, into *S. miltiorrhiza* seedlings. They found that under both laboratory and field conditions, this strain significantly promoted plant growth and increased the content of salvianolic acid B. Zhou, Tang & Guo (2018) introduced another stem-derived endophytic fungus, *Cladosporium* sp. SM58, into *S. miltiorrhiza* seedlings. SM58 colonized the root epidermal and cortical cells, significantly enhancing plant height, root length, and biomass. It also increased root dry weight, total phenolic acid content, and salvianolic acid A content by 68%, 47%, and 11%, respectively. In a broader study, Lin (2018) found that multiple endophytic fungi from generally enhanced seedling biomass *in vitro*. Under pot conditions, most strains promoted both aerial and underground growth, with improved fresh and dry weights of the medicinal root parts. These benefits were linked to fungal regulation of photosynthesis and carbon-nitrogen metabolism, though mechanistic details differed across strains. Furthermore, these nine endophytic fungi exhibit distinct regulatory effects on the biosynthesis of key bioactive compounds in *S. miltiorrhiza*, including rosmarinic acid, salvianolic acids A and B, caffeic acid, as well as the tanshinones (dihydrotanshinone, tanshinone I, tanshinone IIA, and cryptotanshinone) (Lin, 2018). Jiang et al. (2019) inoculated the tanshinone-producing endophytic fungus U104 into young *S. miltiorrhiza* seedlings. After 20 days, the tanshinone content in the inoculated seedlings was significantly higher compared to the control group. Transcriptome sequencing revealed that infection by the endophytic fungus led to upregulation of 14 key enzyme genes involved in the tanshinone biosynthesis pathway, including DXS, DXS2, DXR, HMGR3, AACT, MK, PMK, GGPPS2, GPPS, KSL, IDI, IPII, FDPS, and CPS. Zhai (2019) demonstrated in a field trial that applying the endophytic fungus D16 as a microbial fertilizer significantly enhanced *S. miltiorrhiza* root growth, increasing fresh weight by 1.63-fold and dry weight by 1.42-fold, while also elevating cryptotanshinone, tanshinone I, and total tanshinone content by 2.07-, 1.89-, and 1.88-fold, respectively. Xie et al. (2023) further confirmed that D16 promotes tanshinone accumulation by activating ABA and SA signaling pathways in the roots. Studies demonstrate that endophytic fungi enhance the growth and coordinately boost the accumulation of both phenolic acids and tanshinones in *S. miltiorrhiza* by regulating host photosynthesis and carbon-nitrogen metabolism, activating ABA and SA signaling pathways, and upregulating key biosynthetic genes.

Zhai et al. (2017) also applied the endophytic fungus D38 of *S. miltiorrhiza* as a microbial fertilizer in the cultivation of *S. miltiorrhiza* seedlings. The results demonstrated that the D38 microbial fertilizer not only suggestively promoted plant growing but also markedly improved the accumulation of tanshinones and salvianolic acids. Chen et al. (2021c) demonstrated that the endophytic fungus *Mucor circinelloides* DF20 strain, isolated from *S. miltiorrhiza* roots, produces IAA and siderophores *in vitro*, thereby exhibiting growth-promoting activity. Upon inoculation into *S. miltiorrhiza* seedlings, DF20 strain colonized intercellular spaces and cell junctions, establishing a stable and non-invasive symbiotic relationship. The endophytic colonization markedly induced expression of CYP76AH1, a pivotal gene regulating cyclization in tanshinone biosynthesis, consequently elevating both production and root deposition of tanshinones. Particularly, tanshinone IIA accumulation attained concentrations of 4.630 ± 0.342 mg/g (Chen et al., 2021c). The aforementioned studies indicate that endophytic fungi in *S. miltiorrhiza* effectively promote host plant growth and synergistically enhance the accumulation of its components through root colonization, secretion of growth-promoting substances, and specific upregulation of key genes involved in tanshinone synthesis.

#### Promoting the growth and metabolite accumulation in *E. cortex*

Zhang (2003) investigated the effects of co-cultivating *E. cortex* tissue culture seedlings with two endophytic fungi, *Aspergillus versicolor* Z24 and *Cladosporium halotolerans* Z27, isolated from *E. cortex* seeds. The study found that compared to the control group, the plant height, leaf color, root length, and dry matter accumulation of the seedlings were significantly enhanced. Further inoculation of these fungi into soil-grown seedlings produced consistent results. Additionally, the application of a single fungal strain improved the activity of antioxidant enzymes in the seedlings and reduced malondialdehyde content. These results demonstrate that the two endophytic fungal strains can effectively stimulate plant growth while improving stress tolerance (Zhang, 2003). Zhang et al. (2017b) discovered that among the 90 endophytic fungi isolated from *E. cortex*, 37 strains exhibited the capability to produce IAA. Notably, eight of these strains demonstrated relatively high IAA yields, indicating their potential for development as bio-fertilizers. Li et al. (2023) discovered that the endophytic fungal strain Y232, isolated from *E. cortex*, secretes zeatin (0.14 μg/mL), IAA (0.88 μg/mL), and salicylic acid (0.53 μg/mL), demonstrating strong plant growth-promoting activity. Treatment with Y232 cell suspension significantly enhanced germination rate, shoot and root length, and fresh and dry weight of both *E. cortex* and maize seedlings compared to the control group. Additionally, the height, biomass, and stem length of potted *E. cortex* plants were notably increased. Furthermore, Y232 upregulated the expression of key genes in the phenylpropanoid biosynthesis pathway, leading to significantly elevated levels of 12 characteristic compounds—including aucubin, geniposidic acid, protocatechuic acid, chlorogenic acid, and caffeic acid—in the leaves of *E. cortex* (Li, 2023). These studies collectively demonstrate that endophytic fungi derived from *E. cortex* significantly promote host plant growth, enhance stress resistance, and boost the accumulation of bioactive metabolites through multiple mechanisms, including phytohormone secretion and regulation of key biosynthetic pathways.

#### Promoting the growth and metabolite accumulation in *G. elata*

Yu et al. revealed that the endophytic fungus *Mycena purpureofusca* JFGL-06, isolated from the tubers of *G. elata*, significantly increased the concentrations of carbon (C), nitrogen (N), sodium (Na), and magnesium (Mg) in the surrounding soil. The fungal inoculation substantially improved soil microbiome structure and functional potential, notably increasing the prevalence of key bacterial (*e.g.*, Acidobacteria) and fungal (*e.g.*, Trichoderma) taxa. Additionally, it increased the abundance of functional genes associated with nucleotide metabolism and energy metabolism pathways, thereby effectively improving the quality of *G. elata* (Yu et al., 2023). Yang et al. (2024a) and Yang et al. (2024b) discovered that the endophytic fungus *Scorodonius mycetinis* GYGL-1, derived from the tubers of *G. elata*, substantially improves germination rates and developmental progression in *G. elata*. Studies demonstrate that endophytic fungi from *G. elata* promote host germination and growth by improving the soil microenvironment and optimizing microbial community structure and function.

### Renewable green drug resources

In the context of slow natural regeneration of medicinal plants, low extraction rates of bioactive compounds, and biodiversity loss due to excessive human collection, The strategic application of medicinal plant-associated endophytic fungi has emerged as an effective strategy to protect rare medicinal plant species and facilitate large-scale production of bioactive pharmaceutical ingredients. Moreover the numerous active components of Qin medicine having been discovered in endophytic fungi of Qin medicine (Table 4), some endophytic fungi of Qin medicine also could produce other drugs.

Li et al. (2016b) screened *Phoma glomerata* D14 from *Salvia miltiorrhiza*, which produced ergosterol at a yield of 10 mg/g, demonstrating significant potential for the low-cost industrial production of pharmaceutical-grade ergosterol. Zhao (2020) isolated an antibiotic, iquinosporin, from the endophytic fungus *Chaetomium globosum* in *S. miltiorrhiza*. This marketed antibiotic exhibits significant antibacterial activity against a range of drug-resistant bacteria. Zhang et al. (2016) identified a new compound, 6-O-demethyl-4-dehydroxyaltersolanol A, and three known compounds-4-dehydroxyaltersolanol A, altersolanol B, and chermesinone B-from the culture medium of the endophytic fungus *Nigrospora* sp. YE3033 associated with *Aconitum carmichaeli*. The tested compounds displayed significant antiviral activity against influenza A/Puerto Rico/8/34 (H1N1), showing half-maximal inhibitory concentrations (IC50) ranging from 0.80 to 8.35 µg/mL. Among these, chermesinone B exhibited particularly favorable pharmacological properties, demonstrating selective antiviral efficacy (IC50 = 2.59 µg/mL) coupled with minimal cellular toxicity (IC50 = 184.75 µg/mL), suggesting its viability as a lead compound for anti-influenza therapeutic development. Chen et al. (2021d) identified the anti-HIV active compound flazine from the endophytic fungus *Trichoderma* sp. FZ-31 isolated from the roots of *Aconitum carmichaelii*.

### Biological transformation

Biological transformation *via* microorganisms to achieve the biosynthesis of novel structural compounds or the efficient production of compounds that are challenging to synthesize chemically is a research focus in the field of biomedicine. Multiple endophytic fungi from *E. cortex* and *Astragalus membranaceus* have been identified as having significant potential to serve as hosts for biotransformation.

Multiple endophytic fungi from *E. cortex* show significant biotransformation activity toward isosteviol, highlighting their potential for producing novel derivatives (Chen et al., 2011). Through microbial fermentation of mung beans using *Phomopsis* sp. XP-8 (isolated from *E. cortex* bark), Zhang et al. (2015a) successfully produced three lignan compounds: pinoresinol diglucoside, its monoglucoside derivative, and the aglycone pinoresinol. The study further demonstrated that mung bean polysaccharides play a crucial role in promoting the biosynthesis of pinoresinol monoglucoside. In a subsequent investigation, they elucidated the biosynthetic pathway of pinoresinol diglucoside, and pinoresinol monoglucoside by *Phomopsis* sp. XP-8: glucose is first converted to phenylalanine, which is then transformed into cinnamic acid, followed by p-coumaric acid, ultimately leading to the formation of piceatannol monoglucoside and piceatannol diglucoside (Zhang et al., 2015b).

Other studies have demonstrated that endophytic fungi isolated from *Astragalus* exhibit promising potential in biotransformation applications. Specifically, researchers identified four endophytic fungal strains—*P. roseopurpureum, A. eureka, N. hiratsukae*, and *C. laburnicola*—that are capable of biotransforming four saponin compounds found in Astragalus plants, resulting in the generation of 66 novel compounds (Feng & Cui, 2024). Ma et al. (2020) utilized the endophytic fungus *Penicillium* sp. R3 isolated from the roots of *S. baicalensis* to ferment and biotransform baicalin, successfully producing oroxylin A with a conversion rate of 42.44% and a fermentation yield of 177 mg/L. This study offers a novel approach for the development and application of oroxylin A. Further investigations have confirmed that the endophytic fungus *P. decumbens* f3-1 from *S. baicalensis* roots, when used to ferment the roots of *Astragalus membranaceus*, can mediate the metabolic transformation of wogonoside into wogonin (91.0% yield). This process significantly enhances the anti-acne activity of *S. baicalensis* root extracts (Zhu et al., 2020). Another study employed endophytic fungus LZY9 from Salvia miltiorrhiza to ferment its stem and leaf waste for cellulase production, achieving an enzyme activity of 13.417 IU/g under optimized conditions (Wang et al., 2021b). These studies collectively indicate that endophytic fungi can efficiently catalyze the transformation of specific plant components, not only generating novel compounds but also enhancing the yield or bioactivity of target products. Furthermore, they offer a feasible approach for the enzymatic conversion and utilization of plant waste resources, highlighting their broad application prospects in natural product development and green biomanufacturing.

### Bioremediation

Several studies have demonstrated that certain endophytic fungi associated with Qin medicinal plants hold promise for bioremediation and biodegradation applications, playing a significant role in environmental treatment. Cao et al. (2023) screened 23 endophytic fungal strains from the roots of *Polygonatum kingianum* plants and identified eight strains belonging to *Cladosporium spp.* that exhibited tolerance to arsenic (As) and cadmium (Cd). Under heavy metal stress, these fungi enhanced antioxidant enzyme activity and increased the levels of antioxidant compounds, while also immobilizing metal ions within their mycelia. This research provides valuable insights for soil bioremediation efforts. Zhang et al. (2017a) discovered an endophytic fungus, *Aspergillus tamarii* AL06, isolated from *Astragalus membranaceus*, which achieved a formaldehyde degradation rate of up to 79.2% during its logarithmic growth phase, indicating its potential for industrial wastewater purification.

## Conclusion and Future Challenges

Authentic Chinese medicinal materials, known for their superior quality and high reputation in specific regions, represent the pinnacle of traditional Chinese herbal resources. Qin medicine, as a crucial component of China’s authentic medicinal materials, has been the subject of extensive research into its pharmacological activities and cultivation practices. The high-quality development of the Qin medicine industry has emerged as a key issue in the regional advancement of TCM. Plant endophytic fungi, which are widely present in medicinal plants, exhibit rich biodiversity and play a significant role in enhancing host plant health. These fungi-derived secondary metabolites demonstrate tripartite bioactivity (anticancer, antioxidative, antimicrobial) with promising applications spanning therapeutic development, crop protection, and functional food production. Current research on endophytic fungi in Qin medicine primarily focuses on several key areas: diversity of endophytic fungi, diversity of secondary metabolites, exploration of bioactive and structurally novel secondary metabolites, and the employment of fungal endophytes for biocontrol purposes. Research on endophytic fungi in Qin medicinal plants currently concentrates on three key aspects: analyzing their biodiversity including species composition, geographical distribution and colonization preferences; exploring secondary metabolites with bioactive properties or novel chemical structures; and investigating their potential applications in biological control as biofertilizers or biopesticides. However, significant research gaps remain; most studies have been restricted to culturable fungal species from medicinal plant parts while overlooking other tissues. More importantly, the fundamental symbiotic mechanisms between host plants and endophytes, along with how these fungi regulate plant growth processes, require much deeper investigation to advance our understanding of these complex biological interactions.

The diversity of endophytic fungi in Qin medicinal plants is significantly influenced by geographic distribution, colonization sites, and cultivation environments. However, existing studies predominantly rely on traditional isolation and cultivation methods, leading to the omission of unculturable fungal communities. Additionally, differences in fungal communities across various tissues have not been systematically compared. Future research should integrate metagenomics and single-cell sequencing technologies to comprehensively analyze the endophytic fungal communities in Qin medicinal plants. Furthermore, it is vital to investigate how environmental aspects drive fungal distribution to provide a basis for optimizing cultivation strategies.

Endophytic fungi from Qin medicinal plants can synthesize bioactive compounds identical to their hosts or novel structures, offering a sustainable alternative for the conservation of rare medicinal plants. However, their industrial application faces two major bottlenecks: (1) metabolic attenuation due to repeated subculturing, and (2) unclear biosynthetic mechanisms, hindering targeted regulation. These issues collectively lead to poor reproducibility and scalability in industrial production. The unpredictable attenuation of metabolite yield upon repeated subculturing (reproducibility challenge) and the difficulties in scaling up fermentation processes without compromising yield (scalability challenge) are major obstacles that need dedicated research. Future efforts must therefore not only aim to elucidate mechanisms but also to develop robust processes that ensure consistent output. Future research should employ multi-omics technologies to identify key genes in secondary metabolic pathways, enhance yield through co-culturing, gene editing, or medium optimization, and develop fungal-plant interaction models to decipher the signaling mechanisms that induce metabolite synthesis. Therefore, there is an urgent need to shift Qin medicinal fungi research from “discovery” to “mechanistic elucidation” to facilitate practical applications.

The potential of endophytic fungi from Qin medicinal plants in enhancing stress resistance and promoting growth has been confirmed, yet their practical application in cultivation remains limited. Key obstacles include: (1) insufficient field trials to validate efficacy, (2) unclear mechanisms of action, and (3) immature techniques for large-scale cultivation and application. Furthermore, several translational hurdles remain largely unaddressed. First, the scalability of laboratory protocols to industrial-level production poses significant challenges in maintaining fungal viability and metabolic consistency. Second, the regulatory framework for the commercial use of endophytic fungi as biopesticides or biofertilizers is still evolving, creating uncertainty and barriers to market entry. Finally, achieving consistent and reproducible efficacy of bioactive compound production under field conditions, with its complex and variable environment, remains a critical unsolved problem that limits reliable application.

Research on endophytic fungi in Qin medicinal plants should not remain confined to basic studies but must progress toward industrialization. Currently, there is an urgent need to establish a standardized strain repository and metabolite database for Qin medicinal endophytic fungi to consolidate resources. Technologically, integrating synthetic biology and fermentation engineering could enhance metabolite yields. Furthermore, promoting collaborative “microbe-Chinese medicine” R&D initiatives is essential. Finally, researchers, enterprises, and government bodies should work in synergy to translate laboratory findings into practical cultivation applications, ultimately achieving dual improvements in both the quality and yield of Qin medicinal products. This field presents not only significant challenges but also transformative opportunities to reshape traditional medicinal production paradigms.

## Supplemental Information

10.7717/peerj.20487/supp-1 Supplemental Information 1 A comprehensive and systematic review of the diversity of endophytic fungi associated with Qin medicinal plants

10.7717/peerj.20487/supp-2 Supplemental Information 2 Novel secondary metabolites produced by endophytic fungi of Qin medicine plants

## Additional Information and Declarations

### Data Availability

The following information was supplied regarding data availability: This is a literature review.
