# Supplementary figures and images for: Endophytic fungi and their secondary metabolites in Qin medicine plants: a comprehensive review of diversity, function, and application
Source: PeerJ. 2026 Jan 9;14:e20487. doi: 10.7717/peerj.20487 (PMC12794636; doi:10.7717/peerj.20487)

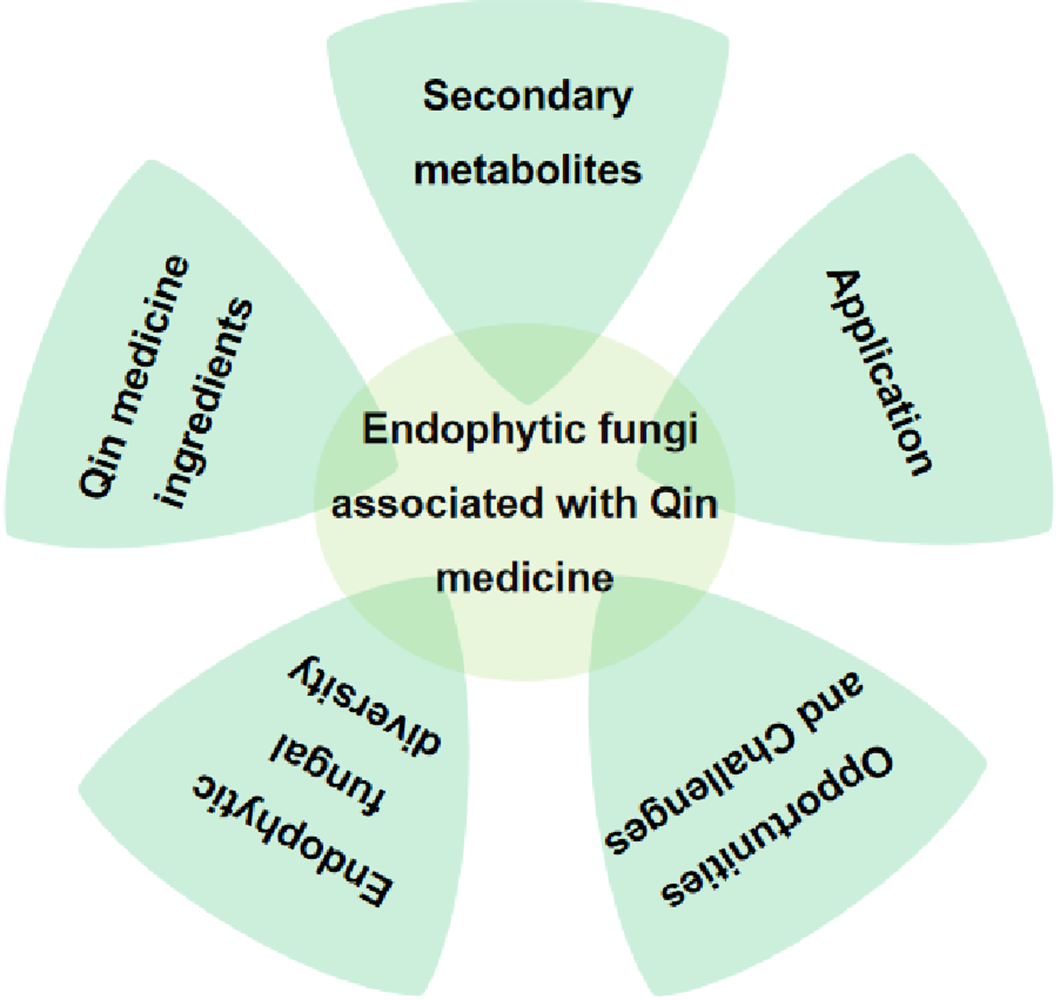

Supplement: Supplemental Information 1 [file peerj-14-20487-s001.png]

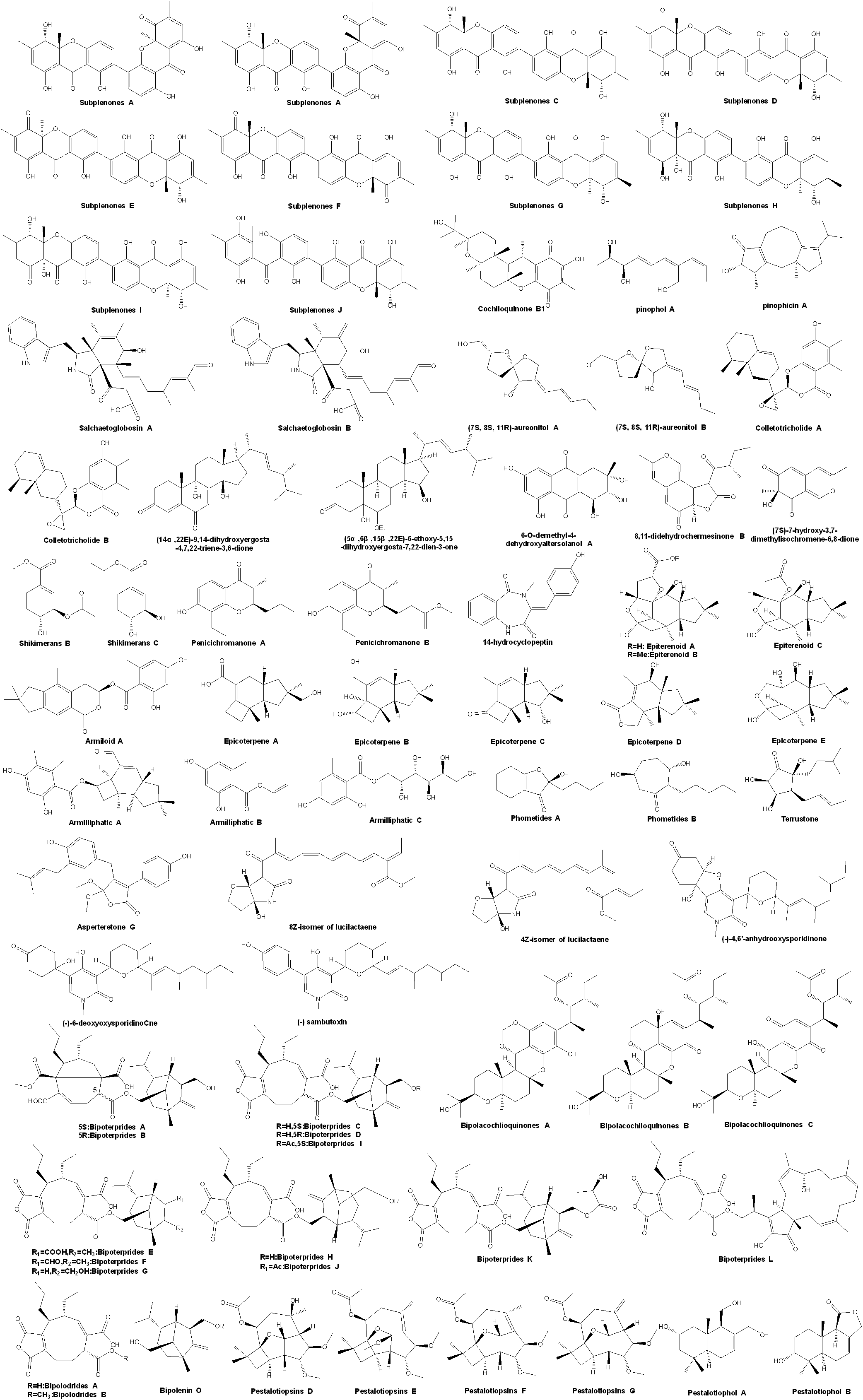

Supplement: Supplemental Information 2 [file peerj-14-20487-s002.png]
